# Improved citric acid-derived carbon dots synthesis through microwave-based heating in a hydrothermal pressure vessel†

**DOI:** 10.1039/d2ra06420k

**Published:** 2022-11-11

**Authors:** Jorns M., Strickland S., Mullins M., Pappas D.

**Affiliations:** Department of Chemistry and Biochemistry, Texas Tech University Lubbock TX USA d.pappas@ttu.edu

## Abstract

Carbon dots (CDs) are a diverse and wide-reaching field of study which encompasses multiple scientific disciplines. This, along with their extensive applications, calls for the need to continually seek to improve their synthesis efficiency. To this end, we focused upon the effects of altering the heating method on the characteristics and quality of the final CD product. In our approach, two different versions of heating techniques were used including the common oven-based hydrothermal method and a microwave (MW)-assisted hydrothermal method. The MW-assisted heating involved the implementation of a pressure vessel (similar to oven hydrothermal vessel) for improved yield of luminescent CDs. We found that this MW method yielded CDs of bright intensity with comparable shape, size, and dispersity to that produced using conventional hydrothermal methods. Specifically, the CDs average particle diameters were determined to be between 1.8 and 2.0 nm for either method. The MW method also demonstrated certain advantages over the oven method in terms of required reaction time and particle yield. The total reaction and cooldown time was reduced by ∼75% (with ideal MW heating time) and an estimation of the average CDs particle density revealed that the MW method was able to produce approximately 1.2 times more particles than the oven method in less time. Correcting for the same processing time, larger yields are available in microwave synthesis. We were able to also demonstrate that both versions of synthesized CDs can serve as viable fluorescence probes for *in vitro* imaging experiments due to the fact that, under the determined working conditions, their fluorescence emission is significantly greater than that of cell autofluorescence [oven: *p* = 1.47 × 10^−27^ (blue), *p* = 6.98 × 10^−4^ (green); 60 second MW: *p* = 7.59 × 10^−10^ (blue), *p* = 3.03 × 10^−4^ (green)] and the CDs were not cytotoxic. This rapid, simple synthesis approach can be applied to other forms of carbon dots and serves as a high-yield alternative to hydrothermal synthesis.

## Abstract 2

Carbon dots (CDs) synthesis from citric acid, ethylenediamine, and formamide by microwave-assisted hydrothermal carbonization in a pressurized vessel.

## Graphical abstract

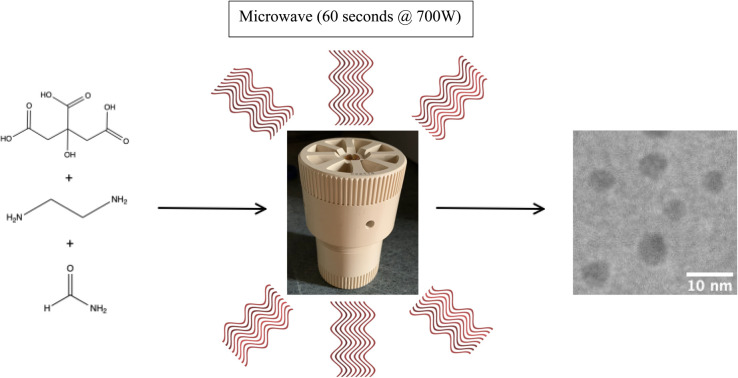

## 1. Introduction

CDs are a specific subgroup of fluorescent nanoparticles which provide multiple benefits that can be utilized for various applications and types of experiments. As their name would suggest, these particles are mainly composed of carbon but also can have additional elements present as dopants or on their surface in the form of surface functional groups.^1–3^ The fact that these groups are almost exclusively hydrophilic as well as the relatively small size of CDs (<10 nm diameter) facilitates the biocompatibility of this material.^1,4,5^ Possibly the most advantageous characteristic of CDs is in their ability to fluoresce with relatively high intensity upon excitation which allows for their potential use in fluorescence microscopy for bioimaging, sensing, and other similar applications. The fluorescence behavior of CDs is not a well understood process and has been observed to be influenced by various physical and chemical characteristics of the particles themselves as well as the surrounding medium. Fluorescence emission of CDs has been reported from the blue region of the visible spectrum to the near-IR region,^1,6–11^ with resistance to photobleaching.^12,13^

**Fig. 1:**
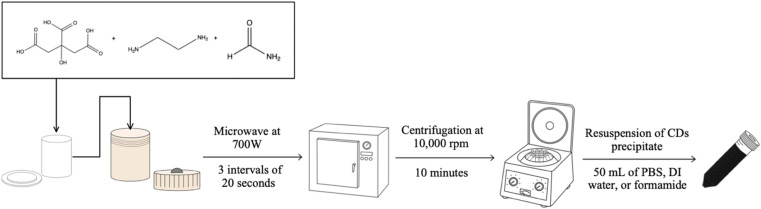
Diagram portraying the steps required to produce CDs by our microwave-assisted hydrothermal method from citric acid, ethylenediamine, and formamide. Each step in the synthesis process introduces opportunities for modification of the final product when exchanging for alternate methods (such as heating by an oven, or purifying by dialysis, *etc.*).

The manufacturing of CDs can be classified into two major categories: top-down or bottom-up synthesis. Top-down methods utilize larger pure carbon materials such as carbon black, soot, graphene, *etc.*, as the carbon source which upon heating is made to fragment into the nano-sized CDs.^1,14^ Bottom-up methods instead rely upon smaller organic chemicals such as citric acid, urea, malic acid, or even animal and plant products or biowaste.^15–17^ Upon the application of heat on these smaller chemicals, the weakest bonds in the structure will break to allow the molecular fragments to reform into the comparatively larger CDs particles. Any functional groups present in the original organic chemical's structure can be retained as surface groups on the CDs, as indicated previously. Each classification of CDs synthesis method provides different benefits, but for our purposes, we chose to focus upon manipulation of the bottom-up rather than the top-down method since it can be less time and energy-consuming and can also be conducted without using highly toxic or hazardous materials. The process for synthesizing CDs allows for easy modification of the final product since each step offers a selection of viable options from the choice of starting materials to final purification step or steps (Fig. 1). Therefore, through modification of the synthesis methodology, we are able to produce a particular type of CD to suit a specific purpose.^18^

The choice of heating method and parameters, such as time and temperature, can impact both composition and yield of CDs.^17,18^ Microwave heating can provide important benefits over heating by a convection oven such as shortened reaction time and reduced energy output requirements due to rapid, localized, and uniform heating by the electromagnetic radiation.^1,17,19,20^ However, the final CDs produced by our MW method must be comparable or of better quality than the same type of CDs produced *via* an oven for these benefits to be truly advantageous. Various articles have been published over the validity of MW-based heating for CDs synthesis.^5,15^ The first reported MW method appeared in 2009 by Zhu, *et al.*^21^ This group was able to synthesize saccharide and polyethylene glycol-based CDs after heating (in an open glass beaker) at 500 W for a mere 3 minutes. More recently, Ergüder, *et al.*, have reported a simple, rapid CDs synthesis method *via* the MW using *o*-phenylenediamine and urea.^22^ Their synthesis was also conducted in a glass vessel but with a reaction time of 20 minutes at 800 MW and resulted in CDs with a yellow emission capable of sensing the chemical aflatoxin B1 in solution. A study by Liu, *et al.*, reports an optimized method for synthesizing CDs with wavelength-tunable emission *via* MW irradiation through adjustment of the quantity of precursors.^23^ They determined the ideal synthesis conditions (reaction time, *etc.*) by monitoring changes in quantum yield until achieving peak intensity and found that, with the concentration of precursors used, 15 minutes of continuous irradiation at a temperature of 180 °C was optimal. The researchers went on to compare their CDs to those produced hydrothermally *via* the oven and concluded that the fluorescence intensity of CDs from the oven method was 2.1 times lower. Additional reported MW-based methods performed under atmospheric pressure can be found throughout the current literature.^11,24–29^

The research articles involving MW-assisted synthesis of CDs most commonly use an unpressurized container as the reaction. The atmospheric-pressure heating methodology has fundamentally different mechanisms for CDs formation compared to that of the hydrothermal carbonization *via* the oven since the containment and recondensing of the solvent and change in pressure is critical for the efficiency of the chemical reaction. In order to most accurately isolate and assess the effect that the MW irradiation itself has upon final CDs products when comparing to the hydrothermal method, the pressure differential created between the external environment and internal solution should also be incorporated in the procedure. This reasoning led us to use analogous versions of a hydrothermal pressure vessel for oven and MW heating methods. One example of a similar MW-assisted hydrothermal method in the scientific literature was reported by Singaravelu, *et al.*, which utilized a microwave Pyrex vessel sealed with a silicone cap and enclosed within a hydrothermal reactor (CEM, Discover SP model) and then heated at constant temperature of 200 °C and power of 300 W.^30^ The CDs produced by their methods used citric acid as the carbon precursor along with *m*-phenylenediamine to form nitrogen-doped particles with a yellow or dual emission depending on the ratio of the two chemicals used. Other studies can be found involving similar MW-assisted hydrothermal synthesis of CDs such as that reported by Purbia, *et al.*, using tender coconut water as a carbon source.^31^ These researchers also utilized a sealed glass vessel inside a microwave reactor, however, their exact method for sealing the vessel was not fully elaborated upon. Since a glass vessel was used in these references, there was a pressure limitation with the experiment setup that would ultimately influence the CDs product, however this aspect was not addressed. Additional studies can be found which report successful synthesis of CDs by MW-assisted hydrothermal carbonization.^32,33^ An interesting article by Wang, *et al.*, examines the effects of MW-assisted techniques upon a common top-down method involving reflux of carbon-based materials in nitric acid.^34^ They were able to demonstrate that the incorporation of MW-assisted synthesis techniques resulted in not only a shortened reaction time but also enhancement of the CDs optical characteristics including increased absorption, higher quantum yield, and longer fluorescence lifetimes. Their results could indicate that microwaving itself provides certain benefits over traditional methods in CDs synthesis, however, top-down methods are different chemical processes than bottom-up methods and so their results cannot be used as a direct comparison in our case.

Therefore, in this work we investigated a hybrid hydrothermal synthesis method using MW radiation in a pressurized vessel. We compared our results with hydrothermal methods for citric acid CD synthesis.^35^ Citric acid is a most common choice for carbon precursor in bottom-up synthesis and serves as a good model for manipulating synthesis conditions.^18^ We discovered with the implementation of this microwaving heating method that we were able to produce approximately 1.15 times more CDs of bright fluorescence intensity and similar physical and chemical characteristics to those produced *via* the oven while reducing the total reaction time by approximately 75%.

## 2. Experimental methods

### 2.1 Materials

Citric acid, ethylenediamine, and formamide obtained from Fisher Chemical. 95% concentration of ethanol used in all experiments. Acetone obtained from Macron Fine Chemicals. Leukemia cell line (HL-60) was purchased from the American Type Culture Collection (ATCC), cultured in RPMI 1640 medium (Hyclone) supplemented with 1% penicillin–streptomycin stabilized solution (Sigma-Aldrich) and 10% fetal bovine serum (Hyclone), and incubated at 37 °C at 5% CO_2_. Phosphate-buffered saline (PBS) purchased from Mediatech, Inc. 7-Aminoactinomycin D (7-AAD) purchased from Thermo Fischer Scientific.

### 2.2 Oven-based hydrothermal CDs sample preparation

0.6 g of citric acid was dissolved in 40 mL of formamide and 1.05 mL of ethylenediamine and mixed using a vortex mixer until the solution was homogeneous. The solution was then placed in the Teflon cup of a hydrothermal vessel (Parr Acid Digestion Vessel 4748) then secured in the metal casing per the manufacturer's specifications. The vessel was then placed in an oven preheated to 180 °C for 4 hours. The vessel was allowed to cool for a minimum of 2 hours at which point the solution was filtered using a 0.2 μm pore nylon membrane disk filter and the CDs precipitated out of solution by the addition of 100 mL of acetone. This sample was transferred to Teflon centrifuge tubes (Thermo Scientific Oak Ridge FEP 50 mL centrifuge tubes) to obtain the precipitate by centrifugation after 10 minutes at 10 000 rpm. The CDs precipitate was washed twice using an ethanol/acetone (1 : 1 ratio by volume) mixture then transferred into a Petri dish to dry overnight. CDs were either kept in solid powder form or resuspended in 100 mL of desired solution (DI water, formamide, or PBS) depending on the sample's intended application.

### 2.3 Microwave-assisted hydrothermal CDs sample preparation

0.3 g of citric acid was dissolved in 20 mL of formamide and 0.525 mL of ethylenediamine and mixed using a vortex mixer until the solution was homogeneous. Due to the limited volume of the hydrothermal vessel (Parr Microwave Acid Digestion Vessel 4781), the solution was split in half and then placed in the Teflon cup of the vessel and secured in the hard plastic casing per the manufacturer's specifications. The vessel was then placed in the MW (Panasonic NE-1054F, 1000 W output, medium–high power setting (70% of output)) for 20 second intervals followed by 30 minute cooling periods until the intended total reaction time was attained (40 seconds, 60 seconds, 80 seconds, or 100 seconds). After the final cooling period is completed, the solution was filtered using a 0.2 μm pore nylon membrane disk filter and then the heating, cooling, and filtering process was repeated with the other half of the citric acid solution. At this point, the second half was recombined with the other half of sample to obtain one MW CDs solution. CDs were precipitated out of solution by the addition of 50 mL of acetone. This sample was transferred to Teflon centrifuge tubes (Thermo Scientific Oak Ridge FEP 50 mL centrifuge tubes) to obtain the precipitate by centrifugation after 10 minutes at 10 000 rpm. The CDs precipitate was washed twice using an ethanol/acetone (1 : 1 ratio by volume) mixture then transferred into a Petri dish to dry overnight. CDs were either kept in solid powder form or resuspended in 50 mL of desired solution (DI water, formamide, or PBS) depending on the sample's intended application.

### 2.4 CD characterization

Transmission electron microscope (TEM) images obtained with the Hitachi S-7650 TEM. Scanning electron microscope and energy dispersive X-ray spectroscopy (SEM-EDS) data were obtained from a Zeiss Crossbeam 540 with an Oxford EDS system. Attenuated Total Reflectance-Fourier Transform Infrared (ATR-FTIR) spectra obtained from a Thermo Scientific Nicolet iS10 Smart ITR FTIR with an ATR crystal attachment. Ultraviolet-visible (UV-vis) absorption measurements obtained using an Agilent 8453 UV-vis spectrometer. Fluorescence spectra obtained from an Agilent Cary Eclipse Fluorescence Spectrometer. Fluorescence lifetime data obtained from a Horiba Jobin Yvon Single Photon Counting Controller Fluorohub with a MicroHR Horiba Jobin Yvon spectrometer. Powder X-ray diffraction (XRD) analysis was conducted on solid-state CDs samples using a Rigaku MiniFlex II. A BD FACSCalibur flow cytometer was used to analyze cell samples for biocompatibility experiments.

### 2.5 CD fluorescence intensity *versus* CD solution incubation concentration assessment

CDs suspended in PBS (60 second MW CDs and oven CDs) were sonicated for 30–60 minutes to ensure proper distribution of CDs in solution. After which, 12 vials of approximately 1 mL of HL-60 cells in cell medium were obtained, centrifuged at 4500 rpm for 5 minutes, then their cell pellets were resuspended in their designated incubation solution. This solution varied in type and concentration of CDs solution from 100% to 0% (by volume) with PBS used for dilution. The 0% CDs concentration (100% PBS) served as the control samples to obtain the average intensity of cell autofluorescence. After 1 hour of incubation in these solutions, the vials were recentrifuged at the same parameters to isolate the cell pellet and resuspend in PBS. The recentrifugation and resuspension in PBS steps were repeated two additional times to remove extracellular CDs and debris from the samples. After this sample preparation is complete, each specimen was imaged using a Nikon Eclipse Ti2-A inverted microscope with a Lumencor Light Engine® Sola 80-10247 high-powered LED as the light source and pco.edge sCMOS camera. Multiple images were obtained with a 20× objective lens under white light and with a blue light band-pass filter cube and a green light band-pass filter cube for fluorescence images. The images were then analyzed using ImageJ software to measure the average fluorescence intensities of cells from each sample under each of the three filter cubes.

### 2.6 Biocompatibility experiments

Previously prepared CDs solutions in PBS (60 second MW CDs and oven CDs) were sonicated for 30–60 minutes to ensure proper suspension of CDs. After which, 9 vials containing approximately 1 mL of HL-60 cells in medium were obtained, centrifuged at 4500 rpm for 5 minutes, then their cell pellets were resuspended in their designated incubation solution. 3 vials were suspended in 1 mL of PBS (control samples), another 3 vials were suspended in 1 mL of diluted 60 second MW CDs in PBS (at previously established maximum staining concentration), and the last three vials were suspended in 1 mL of diluted oven CDs in PBS (at previously determined maximum staining concentration). All vials were left to incubate for 30 minutes after which 1 μL of 7-AAD (1 mg mL^−1^ in dimethylsulfoxide) was added to each vial and then incubated another 30 minutes. Then the vials were recentrifuged at the same parameters to isolate the cell pellet and resuspend in PBS. The recentrifugation and resuspension in PBS steps were repeated two additional times to remove extracellular CDs and dye, as well as debris from the samples. After this sample preparation is complete, a small portion of each specimen was imaged using the fluorescence microscope. Images were captured with the 20× objective lens under white light and with the blue, green, and red-light band-pass filter cubes for fluorescence images. The remaining sample volumes were assessed using a flow cytometer to measure the distribution of fluorescence intensities within a 10 000 cell population. The control samples were used to establish the gates on the histogram for which portion of the population are dead by 7-AAD staining while the gates for the live cells were adjusted slightly for each sample since there is variability in their fluorescence intensity by CDs staining. The triplicate trials of each sample were used to perform *t*-tests to determine if the cell viability (% live cells) varied to a statistically significant degree at the applied CDs staining concentration.

## 3. Results and discussion

The intent with the design of the MW-assisted hydrothermal synthesis procedure was to mimic the parameters of the typical oven-based hydrothermal method as close as possible to isolate the heating method as the sole variable of interest. This was easily achievable except in the type of hydrothermal vessel since the version used in the oven consists of a metal casing. The same manufacturer provides alternate versions of the acid digestion vessel meant to be used for the MW that instead utilizes a hard plastic casing over the inner Teflon cup to still allow us to perform hydrothermal carbonization for CDs formation in solution. Due to the limitations of this vessel's materials, the upper pressure limits that can be safely withstood are lower than that of the metal vessel and so the imbedded safety features are modified accordingly for this version. As a result, the reaction time in the MW is strictly limited to short increments as opposed to the continuous heating done in the oven. From experimentation we determined that, for our type of MW used, at the medium–high setting, a maximum of 20 second heating intervals could be conducted with 30 minute cooling periods between heating cycles per the manufacturer's specifications. After 40 seconds of total reaction time in the MW, CDs were able to be produced, therefore, this was established as the minimum time required to synthesize CDs *via* the MW. Additional trials were conducted at increasing reaction times up to a maximum of 100 seconds to obtain an array of MW CDs in order to establish the optimal reaction time for this method.

In terms of reaction and cooling time for MW and oven CDs samples, the MW samples had a significantly lower time requirement. Times for synthesizing CDs in the MW, including the required cooling periods, ranged from 1 hour and 40 seconds to 2 hours 31 minutes and 40 seconds. Alternatively, the CDs synthesized in the oven took 4 hours followed by a minimum of 2 hours of cooling, however, an additional 30–45 minutes of cooling was often required before the metal casing of the vessel could be safely handled, and the inner Teflon cup removed. This demonstrates that the MW method can reduce the required CDs synthesis time by ∼58–85%, which provides tremendous benefit towards expediting research and potentially enhancing the productivity of CDs manufacturing on the larger scale.

**Fig. 2:**
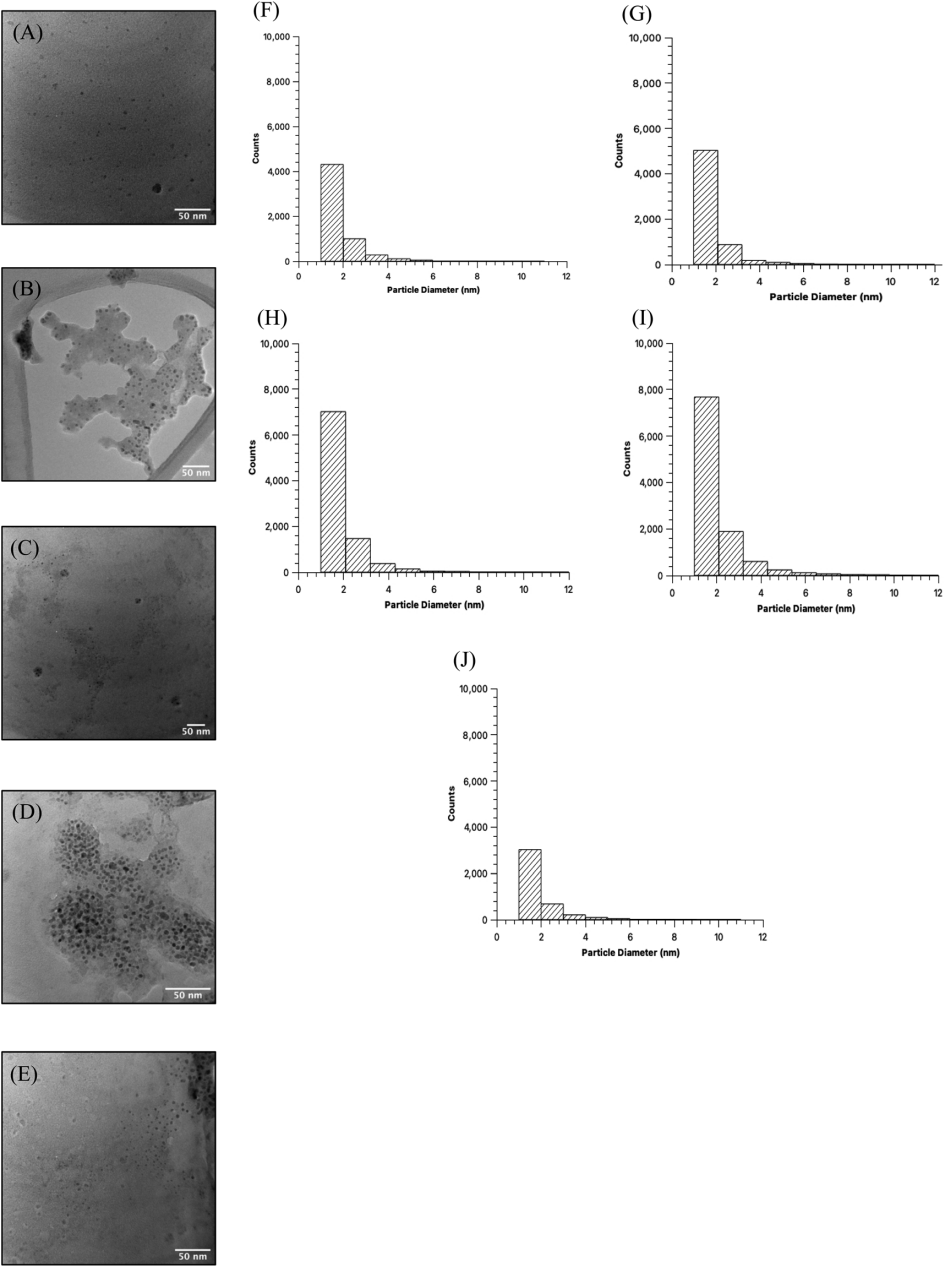
Representative TEM images of CDs samples in PBS and size distribution histograms. From each TEM image, we can observe a certain degree of aggregation of CDs, however, the CDs generally display good dispersion which is consistent across all samples. Particle shapes also appear generally quasi-circular. Images correspond to (A) 40 seconds MW CDs, (B) 60 seconds MW CDs, (C) 80 seconds MW CDs, (D) 100 seconds MW CDs, and (E) oven CDs. CDs sizes were obtained through the particle analysis software on ImageJ program. The analysis was conducted with specifications set to isolate CDs with diameters of 1–10 nm and so the full size distribution is not displayed for these samples. Each histogram corresponds to (F) 40 seconds MW CDs, (G) 60 seconds MW CDs, (H) 80 seconds MW CDs, (I) 100 seconds MW CDs, and (J) oven CDs.

Comparison of the quality of the CDs samples began with high resolution TEM imaging to obtain the visual representation of particles and discern physical characteristics (Fig. 2A–E). Images were obtained for MW CDs and oven CDs suspended in the same solution, in this instance PBS, to avoid any influence that differing solutions may have on particle aggregation. Multiple images were obtained for each CDs sample (see ESI Section A† for all TEM images) and from each representative image it was determined that, visually, the CDs appeared to have similar shape and distribution across all samples. To quantify the degree of similarity in particle shapes the produced CDs were assessed for their relative roundness (scale from 0 to 1 with value of 1 meaning completely circular) using ImageJ software measurements of every sample's TEM images (individual particle measurements can be provided upon request). The analyzer was specified to only include CDs with diameters from 1 to 10 nm to exclude large particles, aggregates, and background noise. With this data, the average roundness of CDs in each sample was calculated as the following: 40 s MW CDs = 0.57 ± 0.16, 60 s MW CDs = 0.56 ± 0.16, 80 s MW CDs = 0.56 ± 0.16, 100 s MW CDs = 0.55 ± 0.17, and oven CDs = 0.56 ± 0.17. From these values we can infer that all samples are of similar shape (more ellipse-like rather than perfectly circular) with the 40 s MW CDs being the closest to circular and the 100 s MW CDs being the least. The diameters of particles in each sample were also assessed from the collective TEM images using the particle analysis feature on ImageJ (Fig. 2). All five samples appear to have CDs with an approximate average particle diameter between 1.8 and 2.0 nm meaning CDs size was not profoundly affected by the type of heating method or reaction time in MW under the analytical methods and specifications used.

Tang, *et al.* report that CDs formation mechanism by a MW-assisted hydrothermal method was theorized to consist of an initial dehydration of a portion of the carbon source molecules to form the starting nuclei composed of a matrix of C=C bonds (CD cores) followed by edge growth on the spherical surface by new C=C bond formation through additional dehydration of source molecules.^36^ In this way the CDs become larger with increasing heating times as edge growth is instigated and amplified, and the growth of CDs is directly dependent on carbon source concentration, microwave power, and heating time. They also propose that the high pressures experienced due to the hydrothermal condition causes the new C=C bond formations to arrange in an orderly and crystalline manner. The experiments performed by this group for this research article were conducted with glucose as the carbon source dissolved in DI water and no added passivating agent so we cannot assume that the formation mechanism which they observed would necessarily be valid for our types of CDs. This formation mechanism is quite similar to that undergone during oven-based hydrothermal carbonization where a “carbon seeding core” initiates particle formation followed by edge growth to increase particle diameter to <10 nm.^37^ The number and size of particles in our samples which were too large to be deemed CDs were not quantified and so the overall trend in particle size with reaction time was not determined.

**Fig. 3:**
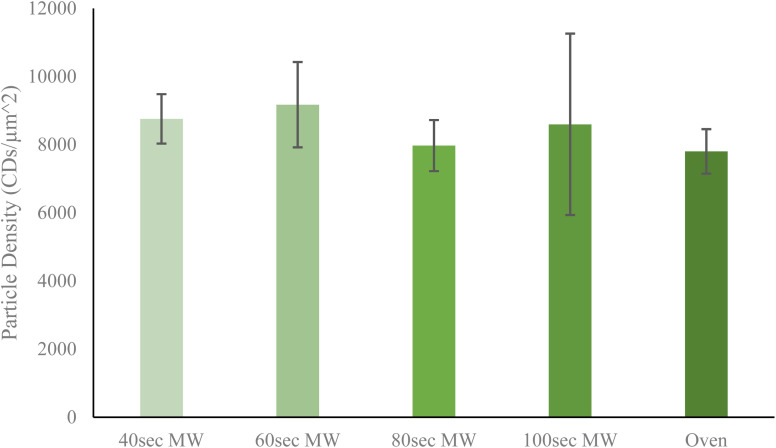
Average particle density as determined from collective TEM images. Reported densities obtained by the particle analyzer feature on ImageJ software as number of particles with specified range of diameter per square micrometer. As reaction time in microwave increases, there is an initial increase then decrease in particle density. The oven CDs sample was revealed to have the lowest particle density. Error bars show standard deviation of particle densities.

Next, the relative yield of particles by each method was determined to discern any trends which might correlate to heating method or heating time. Particle yield is a rather important aspect of CDs synthesis since an ideal method should have the highest possible yield to increase efficiency of resources and time. From Fig. 3, it is shown that of all five CDs samples, the 60 second MW CDs had the highest particle yield at 9005.915 CDs per μm^2^, while the oven sample had the lowest at 7802.932 CDs per μm^2^. It must be noted that these reported densities should not be used to conclude upon precise particle concentrations in sample solution but instead should be used only to compare relative particle yields between each method performed. An overall trend was observed with the MW CDs where particle density initially increased from 40 to 60 seconds of heating then decreased as reaction time was further increased (Fig. 3). This could indicate that when heating by microwaves the CDs are being destroyed, aggregated, or otherwise rendered nonviable after being synthesized at 60 seconds as excess heating is applied. This demonstrates an additional advantage for MW CDs over oven CDs, besides the shortened synthesis time, since fewer synthesis runs would need to be conducted to achieve the same number of CDs. This saves on laboratory resources as well as time available for performing various experimentation. For all subsequent biological experiments conducted with CDs, the 60 second MW sample served as the representative for the ideal or best quality MW sample. To corroborate the data obtained from the TEM images for particle yield per CDs sample, we turned to the standard addition method *via* UV-visible spectrometry measurements (see ESI Section B† for UV-visible spectrometry data). Once we were able to confirm that the CDs samples followed Beer's law in working concentration limits, we conducted the typical standard addition method using diluted CDs solution as the starting sample and undiluted CDs solution as the standard. The resulting calibration curves allowed us to estimate an arbitrary value for particle concentration with which to compare between samples. A similar trend in particle concentration to that derived from TEM images was observed with this UV-vis data, further supporting that the 60 second MW CDs sample had the highest particle content.

**Fig. 4:**
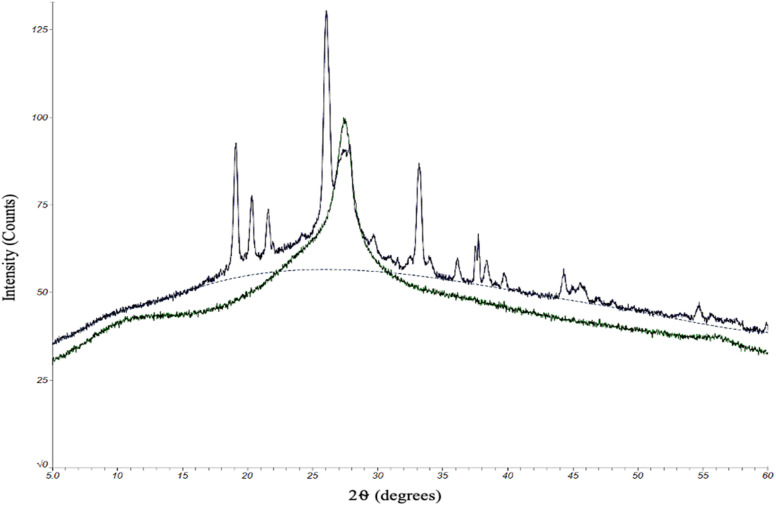
XRD data for solid state CDs produced from the oven method (green) and microwave method (dark blue). The single large graphene peak found in the oven CDs sample data near 27° indicates that the particles are crystalline and likely made up of stacked layers of graphene with interlayer spacing around 3.3 Å. The 100 s MW sample was used to represent the microwave sample. The microwave sample data most notably has additional peaks present which demonstrate that these CDs have a different crystalline makeup than the oven but still retain the large central graphene peak.

The quality of the CDs samples was also assessed by their chemical characteristics to determine how each kind of CD might differ in degree of crystallinity and doping of electron-rich elements (oxygen and nitrogen). Any differences in chemical composition could ultimately have an effect on the performance of the CDs. To this end, we began with an analysis by powder X-ray diffraction (XRD). The protocol for oven and MW CDs synthesis was followed excluding the final resuspension step in order to produce solid CDs samples for this analytical method (Fig. 4).

Due to practical time constraints, the 100 second MW CDs were used as the representative MW sample since the solid particle yield from the 60 second method was inadequate for this analysis but was not the case for the samples from the highest reaction time. As previously theorized, higher reaction times potentially leads to a greater degree of aggregation and would therefore result in a collection of particles large enough to be seen by naked eye and manually retrieved after the drying stage of the synthesis method. XRD patterns were obtained by scanning a 2*θ* range of 5–60°, step size of 0.02°, and scan time of 6.0 degrees per minute. The X-ray source was Cu Kα radiation with an anode voltage of 30 kV and current of 15 mA. The data produced from the analysis of our standard oven CDs revealed a single large peak around 2*θ* = 27°, which, based upon the chemicals and elements present in the reaction conducted, correlates to a graphene structure.^38,39^ We concluded that these CDs are almost certainly composed primarily of graphene-like layers interconnected to form a highly crystalline structure. The 100 second MW sample also contained the large central graphene peak but there were additional sharp peaks that were not present in the oven sample. Since we were able to confirm that these excess peaks are not contaminants, our interpretation of the resulting data is that the microwaving method had a higher degree of heterogeneity in inter-layer spacing. This could be due to a greater extent of O- and/or N-doping of the carbon network than the oven method which would slightly expand the graphene-like layers (peaks below 2*θ* = 27°),^38–40^ and these doped elements could also display an increased degree of bonding with each other between the interconnected layers. This would shorten the distance between layers forming a more rigid and crystalline structure than what was indicated by the oven sample (peaks above 2*θ* = 27°).^41^ The chemical structure of the two types of CDs, however, would need to be illuminated more completely before concluding upon any of these theories.

**Fig. 5:**
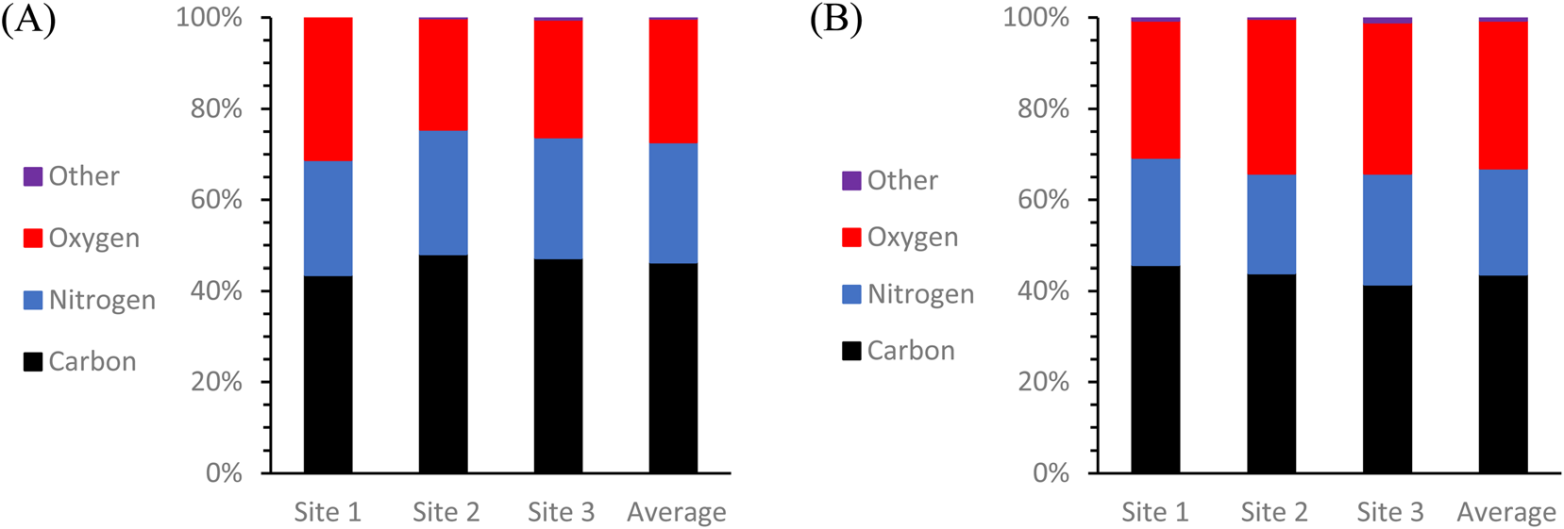
Elemental composition of CDs samples derived by EDS mapping. Graphs depict the weight% of each element detected at each sample location and also the calculated average elemental weight% of (A) oven CDs and (B) 100 second MW CDs solid samples. Both types of CDs are mainly composed of C, O, and N with minimal contamination by other elements (≤1%). Oven CDs consist of an average of 46.3 ± 0.3% of C, 26.4 ± 0.4% of N, and 27.1 ± 0.2% of O. The 100 second MW CDs consist of an average of 43.7 ± 0.3% of C, 23.2 ± 0.5% of N, and 32.4 ± 0.3% of O.

To get a more comprehensive view of the elemental composition of these same solid CDs samples, an additional test was conducted using scanning electron microscopy and energy dispersive X-ray spectroscopy (SEM-EDS) (Fig. 5). All SEM images and EDS map sum spectra can be viewed in ESI Section C.† The resulting EDS data reveals that there was almost exclusively C, O, and N present in both types of CDs with any and all other detected elements totaling less than or equal to 1% of the particle composition. It is most noteworthy that although these two types of CDs were synthesized from identical starting materials, there is 5.3% more O and 3.2% less N in the MW CDs which can indicate a difference in particle formation mechanism from the oven CDs, but this would need to be examined further to draw any conclusions. One possible explanation could be found from examining the difference in temperature changes experienced by the system with each method. The MW method involves a series of rapid heating and cooling cycles while the oven method uses an extended heating and cooling period. Could heating in series rather than in one long period therefore be likened to using a blender in pulse mode rather than simply flipping the “on” switch? Under this premise, the change in particle composition would possibly result from the difference in how the carbon source was broken down with more of the O-containing functional groups being retained from the citric acid in the MW method.

**Fig. 6:**
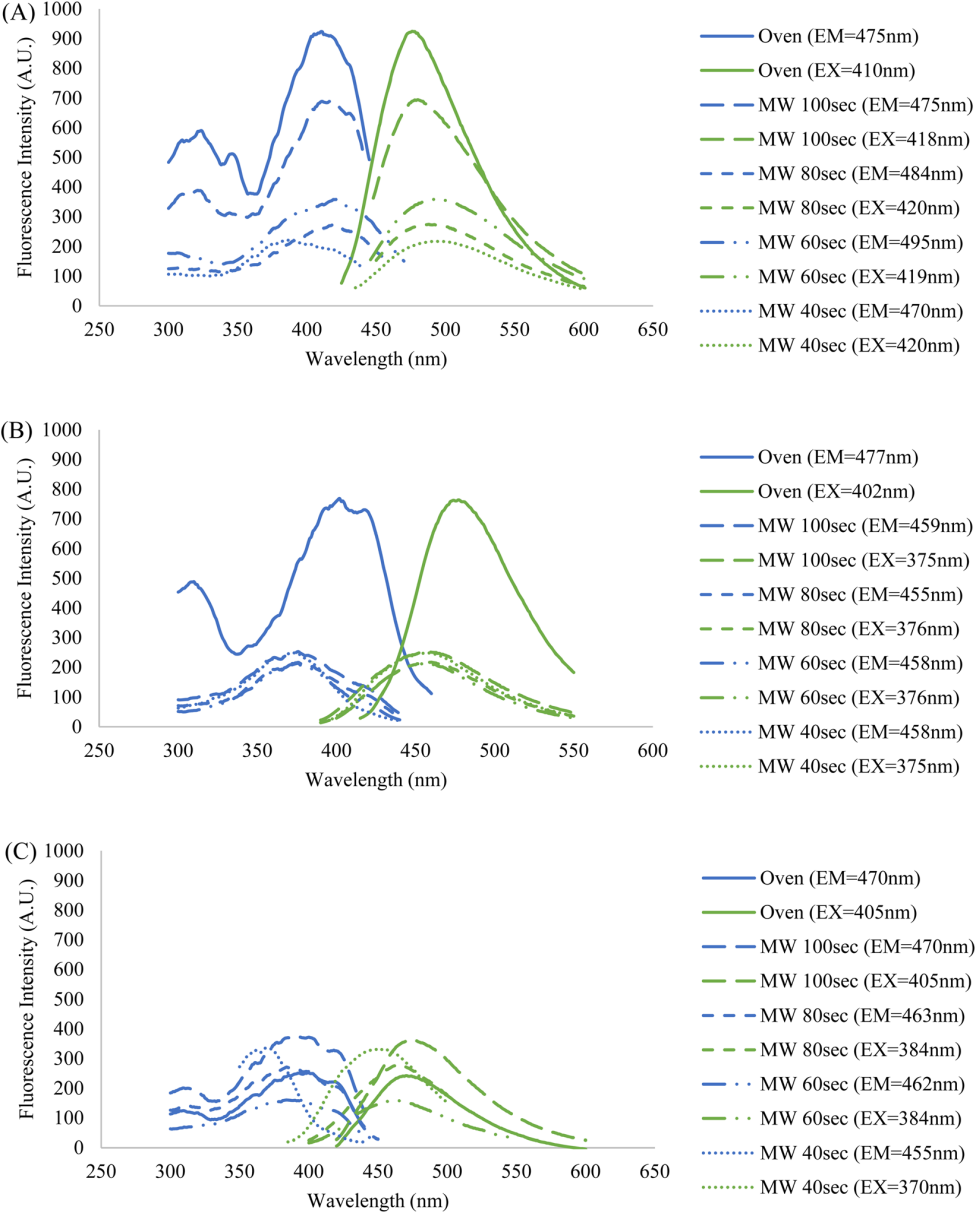
Fluorescence profiles of the five CDs samples resuspended in (A) formamide, (B) DI water, and (C) PBS. Each displayed trendline contains that samples' highest peak fluorescence intensity corresponding to their ideal concentration, and excitation and emission wavelengths (ESI Section E†). The fixed wavelengths used to obtain excitation and emission spectra were chosen based on each sample's maximum fluorescence intensity measured during emission and excitation scans, respectively. The blue spectra indicate an excitation scan at a constant emission wavelength while the green spectra indicate an emission scan at a constant excitation wavelength.

Since the intended application of these CDs samples is in fluorescence microscopy, their fluorescence behavior must be illuminated to compare their potential as imaging probes. The fluorescence profiles of the five samples in three different resuspension solutions were also obtained using a fluorospectrometer. For each sample, a series of measurements were conducted at varying sample concentrations and excitation and emission wavelengths to assess what particular conditions resulted in the peak intensity (ESI Section E†). The collective fluorescence trendlines containing the peak intensities for each solution are reported in Fig. 6.

It was observed that the identity of the resuspension solution has a considerable effect upon the fluorescence behavior of the CDs. Additionally, the wavelengths of excitation and emission are kept fairly constant amongst the five CDs samples in formamide and DI water, but there is a lesser degree of overlap when in PBS. This indicates that the saline solution has a differing effect on fluorescence between and among the CDs samples, particularly for the 40 second MW sample. This is further demonstrated by the discrepancy in fluorescence intensity between the MW samples and oven sample when suspended in PBS and DI water, however, no clear explanation of this phenomenon is available. One crucial observation to be made from the graph containing samples in PBS (Fig. 6, graph (C)) is that the highest fluorescence intensity does not come from the oven sample, but rather a MW sample. The oven sample actually had the second lowest peak excitation and emission intensities. Therefore, from this data we have shown that MW CDs are capable of demonstrating a higher fluorescence intensity than oven CDs under certain conditions but since other characteristics are involved in selecting an ideal imaging probe for biological samples this should not be the determining factor on which type of CDs is best. Since PBS is the medium required to conduct biological experiments, the differences in intensities in this solution *versus* other solutions have a significant impact upon their application in this area of study.

It should be noted that a trend emerges between the five samples within each set with respect to the percent concentrations of CDs solutions utilized to achieve peak intensity. Generally, the MW samples required greater degrees of dilution as reaction time was increased and the oven sample had the highest dilution. This may initially lead one to conclude that greater dilution is required due to a greater density of CDs in solution, however previous data contradicts this notion. Therefore, the most logical interpretation of the fluorescence data would suggest that fluorescence intensity is not directly related to particle counts, or at least not this criterion alone. Certain types of nanoparticles can demonstrate a greater fluorescence intensity when localized together or aggregated than as singular particles.^42,43^ This may be the case in our situation if the oven and higher MW reaction time samples had a greater number of CDs aggregates, but this could not be determined from the TEM images that were obtained. As previously stated, the fluorescence behavior of CDs is a process that is not fully understood and so the reasons behind this trend in fluorescence intensities and excitation and emission wavelengths can be speculated at but would be difficult to form conclusions upon with the analytical methods available to us. Attaining maximum fluorescence intensity must be balanced with maintaining the ideal physical and chemical aspects of the CDs sample for any intended application.

The fluorescence behavior was also examined by measuring each CDs sample's respective lifetime decay since this aspect is directly relevant for fluorescence applications. An exceedingly long lifetime decay of a fluorophore would render it as an impractical imaging probe. The following equation:1
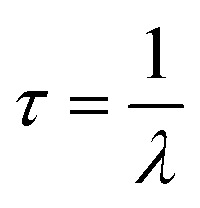
was used to calculate the lifetime decay from the raw data where *τ* is the lifetime of the CDs and *λ* is the decay constant (see ESI Section F† for fluorescence lifetime graphs). The oven CDs were determined to have a lifetime of 7.023 ns while the 60 second MW CDs had a shorter lifetime at 6.145 ns. Both lifetimes are sufficiently short for most fluorescence applications.

**Fig. 7:**
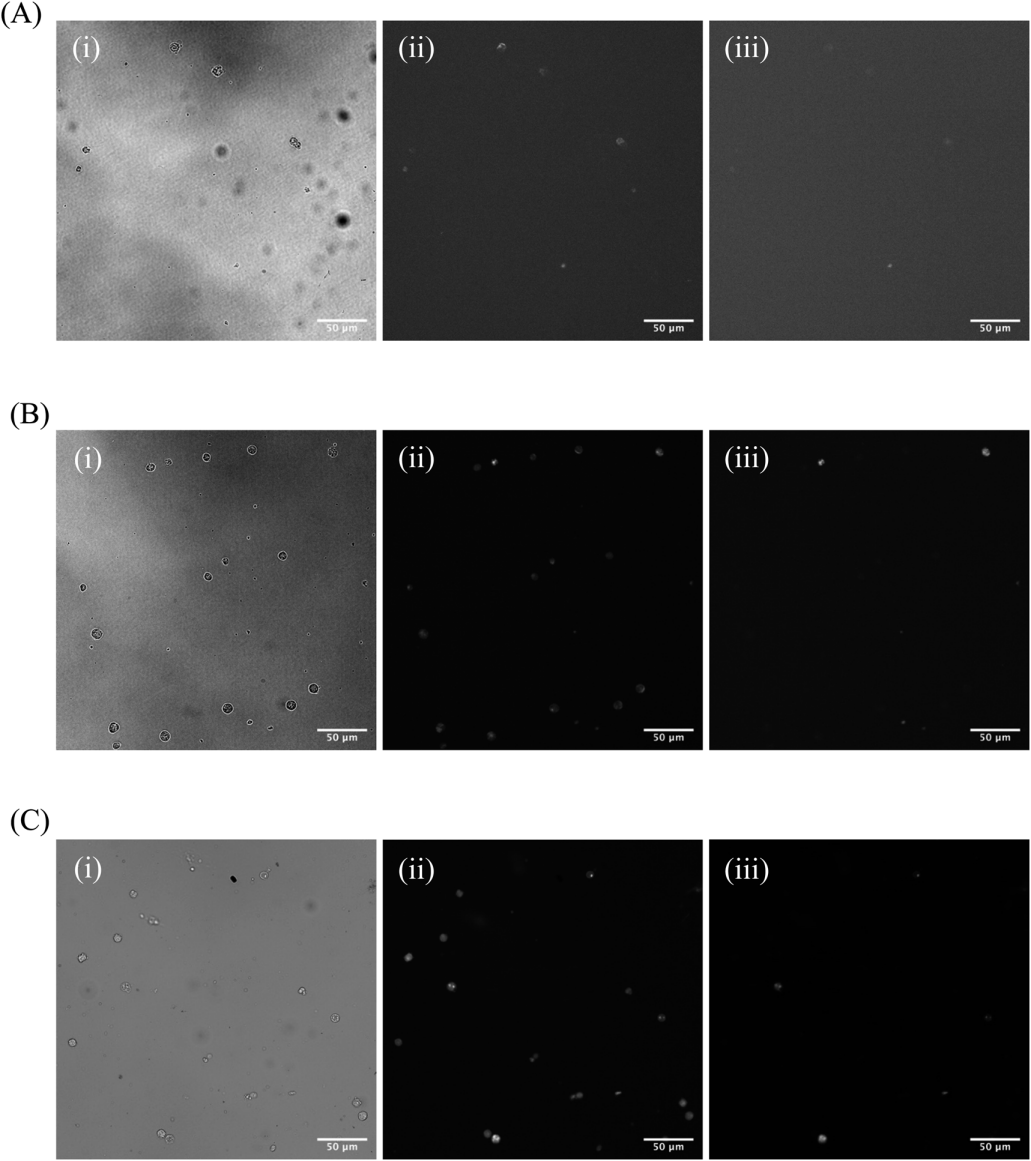
(A) Representative fluorescence image of HL60 cells control sample under (i) white light and the (ii) blue and (iii) green band-pass filters. (B) Representative fluorescence image of HL60 cells stained with 50% concentration of 60 second MW CDs in PBS under (i) white light and the (ii) blue and (iii) green band-pass filters. (C) Representative fluorescence image of HL60 cells stained with 10% concentration of oven CDs in PBS under (i) white light and the (ii) blue and (iii) green band-pass filters.

**Fig. 8:**
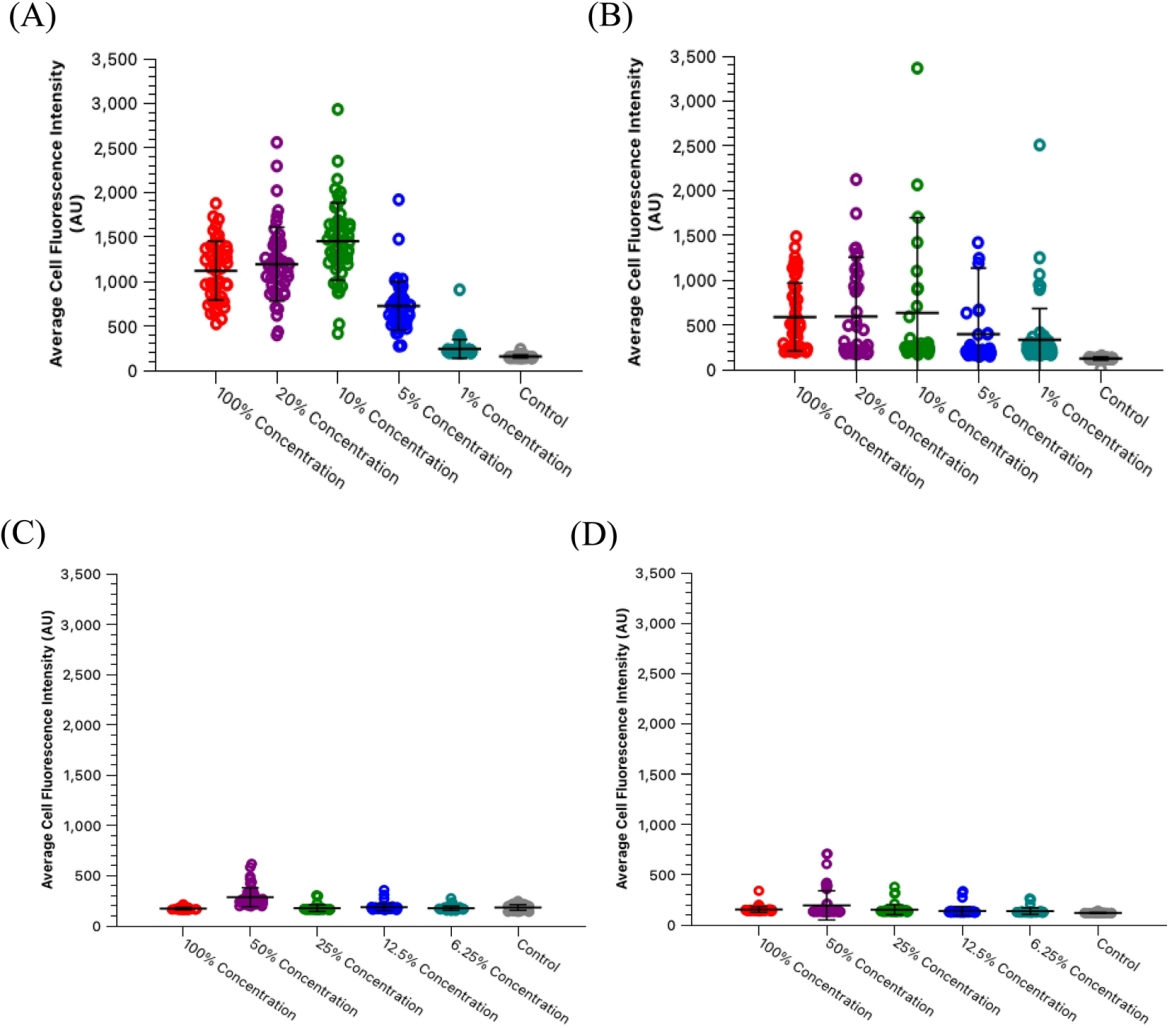
HL60 cell staining concentrations with oven CDs and 60 second MW CDs in PBS *versus* average fluorescence intensities. Each data point displayed on the graphs represents a single cell imaged and then analyzed through ImageJ software. (A) Cells incubated with oven CDs in PBS staining solutions and imaged under blue light. (B) Cells incubated with oven CDs in PBS staining solutions and imaged under green light. (C) Cells incubated with 60 second MW CDs in PBS staining solutions and imaged under blue light. (D) Cells incubated with 60 second MW CDs in PBS staining solutions and imaged under green light. Graphs reveal peak fluorescence intensities for the oven CDs solution at 10% concentration and then 50% concentration for the 60 second MW CDs solution.

One significant application for CDs is in fluorescence microscopy of biological samples as fluorescent probes. For the CDs to be considered as feasible probes, they must demonstrate a fluorescence intensity which is greater than that produced by cell autofluorescence. To this end, we conducted a series of experiments to determine what incubation concentration of the two types of CDs solutions (60 second MW CDs and oven CDs in PBS) resulted in the greatest average cell fluorescence intensity. This concentration would then serve as the maximum concentration required for fluorescence microscopy so long as the CDs intensity proved to be statistically greater than that of cell autofluorescence. The procedure was followed as described in Section 2.5 for preparing HL60 cell samples at a range of CDs solution concentrations. The fluorescence images gathered from these samples and the control samples were analyzed using ImageJ to determine the average intensity of each cell in each frame under each type of filter to calculate the sample's overall average intensity of “stained” and “unstained” cells. Representative microscopic images are shown in Fig. 7 to visually demonstrate that each type of CDs was internalized into the cells. Plots were generated to track change in average intensity with CDs sample concentration (Fig. 8). Individual cell fluorescence intensity measurements can be provided upon request.

Within the range of concentrations tested for each solution, the oven samples had cells with the highest average cell fluorescence intensity when diluted with PBS to a 10% concentration while the 60 second MW sample peaked at the 50% dilution in PBS under both types of light filters. The calculated average intensity for each sample was compared against the average intensity of the control samples by a *t*-test to determine if the intensities were significantly greater than cell autofluorescence. Both types of CDs were revealed to be sufficiently intense to serve as fluorescence imaging probes at these concentrations [oven: *p* = 1.47 × 10^−27^ (blue), *p* = 6.98 × 10^−4^ (green); 60 second MW: *p* = 7.59 × 10^−10^ (blue), *p* = 3.03 × 10^−4^ (green)]. Therefore, cell staining by CDs should be performed at a maximum of 50% concentration with the 60 second MW sample and 10% with the oven sample, however, if these concentrations prove to significantly increase the rate of cell death (cytotoxicity due to excess CDs in solution), this percentage can be decreased so long as the resulting fluorescence intensity is still significantly higher than cell autofluorescence.

**Fig. 9:**
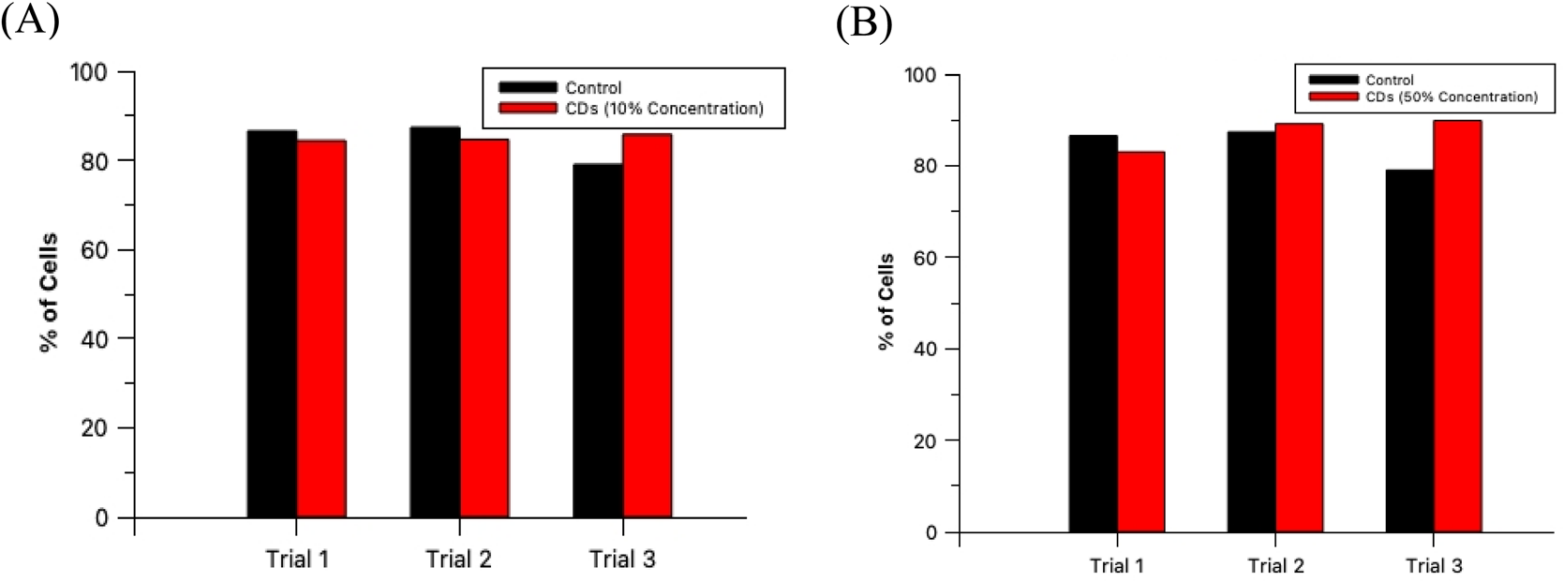
HL60 cell viabilities as the percentage of total sample population for control (black) and test (red) samples in triplicate. Test samples include (A) oven CDs and (B) 60 second MW CDs at their previously established maximum staining concentrations, 10% and 50%, respectively.

The non-toxic nature of CDs in general provides a tremendous benefit over synthetic fluorescent dyes since this allows for extended and extensive *in vitro* fluorescence imaging experimentation. For an imaging agent which severely effects its sample in a harmful way ultimately negates its purpose since it would not provide a true image to the researcher. To verify that these CDs are, in fact, biocompatible at these established concentrations, HL60 cells were prepared and analyzed as described in Section 2.6. A diagram depicting this process is provided in the ESI (Section G)† as well as cell microscopic images for the control and test samples (ESI Section H†) and labeled histograms generated by the flow cytometer (ESI Section I†). The results from this experiment are summarized in Fig. 9. An analysis by *t*-test between the percent of live cells of the 3 control samples and the 3 oven CDs samples with 10% staining concentration showed no statistically significant difference between the two groups (*p* = 0.427). Moreover, the same analytical method was conducted with the 60 second MW CDs samples (3 replicates) stained at 50% concentration and yielded similar results as with the other CDs sample (*p* = 0.273). We can conclude that these MW CDs and oven CDs are biocompatible at the established working conditions and therefore are viable probes for fluorescence imaging of biological specimen.

## 4. Conclusion

In conclusion, we have demonstrated certain critical advantages of MW-assisted hydrothermal CDs synthesis over the traditional oven synthesis method. First and foremost, the significantly reduced reaction time, approximately 75%, and reduced energy requirements of the MW method allow for a more expedient synthesis process. The size, relative shape, and distribution of MW CDs is comparable to that produced by the oven CDs by an assessment *via* TEM images. Specifically, the particle diameters were quantified and found to average between 1.8 and 2.0 nm for all samples which demonstrated that the size of viable CDs (defined as being <10 nm in diameter) was not drastically affected by heating time or method, contrary to literature references. However, some of the reported data did suggest that there may be an increase in aggregation of CDs in solution with longer reaction times in the MW and in the oven, but this could not be directly confirmed in the TEM images. An additional advantage obtained with the MW-assisted method was in the overall yield of CDs particles with the intended size of 1–10 nm diameters which proved to be highest for the MW sample at the 60 second reaction time when compared to all other samples. Additionally, the fluorescence behavior of both types of CDs showed similar peak excitation and emission wavelengths in some of the resuspension solutions used at their optimal concentrations of CDs. Fluorescence intensities differed between versions of CDs and resuspension solution identities but most notably with PBS the oven CDs proved to have a lower intensity than most of the MW CDs samples. While the ideal concentrations of CDs solution for MW and oven samples were established at different percentages in the biological experiments conducted, 50% and 10%, respectively, we were able to demonstrate that both samples were biocompatible with no statistically significant increase in cytotoxicity at their respective working concentrations (*p* = 0.273 for the 60 second MW CDs and *p* = 0.427 for the oven CDs). Therefore, we propose that the MW serves as a viable and more advantageous alternative to the oven to produce better CDs samples more efficiently. Further study should be conducted on these samples to thoroughly evaluate the differences in chemical characteristics of these CDs and discern more information about the complete structure of the particles such as by atomic force microscopy (AFM) and other commonly used analytical techniques. It would be crucial to also assess the localization of CDs in cells to identify any affinities the CDs may have to cell components/structures/types using the super-resolution radial fluctuations (SRRF) program. Additionally, it would be important to also analyze the differing effects upon the final CDs product when modifying other aspects of the synthesis process, such as the choice of starting materials and ratio of components, purification methods, *etc.*, in order to further improve upon the final CDs product for *in vitro* fluorescence microscopy of biological samples for future application.

## Conflicts of interest

There are no conflicts to declare.

## Supplementary Material

RA-012-D2RA06420K-s001

## References

[cit1] Ganguly S., Das P., Banerjee S., Das N. C. (2019). Advancement in science and technology of carbon dot-polymer hybrid composites: a review. Funct. Compos. Struct..

[cit2] Das P., Ganguly S., Agarwal T., Maity P., Ghosh S., Choudhary S., Gangopadhyay S., Maiti T. K., Dhara S., Banerjee S., Das N. C. (2019). Heteroatom doped blue luminescent carbon dots as a nano-probe for targeted cell labeling and anticancer drug delivery vehicle. Mater. Chem. Phys..

[cit3] Barman M. K., Jana B., Bhattacharyya S., Patra A. (2014). Photophysical Properties of Doped Carbon Dots (N, P, and B) and Their Influence on Electron/Hole Transfer in Carbon Dots–Nickel (II) Phthalocyanine Conjugates. J. Phys. Chem. C.

[cit4] Li L., Li Y., Ye Y., Guo R., Wang A., Zou G., Hou H., Ji X. (2021). Kilogram-Scale Synthesis and Functionalization of Carbon Dots for Superior Electrochemical Potassium Storage. ACS Nano.

[cit5] Sharma A., Das J. (2019). Small molecules derived carbon dots: synthesis and applications in sensing, catalysis, imaging, and biomedicine. J. Nanobiotechnol..

[cit6] Chen Y.-X., Lu D., Wang G.-G., Huangfu J., Wu Q.-B., Wang X.-F., Liu L.-F., Ye D.-M., Yan B., Han J. (2020). Highly Efficient Orange Emissive Graphene Quantum Dots Prepared by Acid-Free Method for White LEDs. ACS Sustainable Chem. Eng..

[cit7] Wang T.-Y., Chen C.-Y., Wang C.-M., Tan Y. Z., Liao W.-S. (2017). Multicolor Functional Carbon Dots via One-Step Refluxing Synthesis. ACS Sens..

[cit8] Lu S., Sui L., Liu J., Zhu S., Chen A., Jin M., Yang B. (2017). Near-Infrared Photoluminescent Polymer-Carbon Nanodots with Two-Photon Fluorescence. Adv. Mater..

[cit9] Ding H., Wei J.-S., Zhang P., Zhou Z.-Y., Gao Q.-Y., Xiong H.-M. (2018). Solvent-Controlled Synthesis of Highly Luminescent Carbon Dots with a Wide Color Gamut and Narrowed Emission Peak Widths. Small.

[cit10] Wang W., Zhang Q., Zhang M., Liu Y., Shen J., Zhou N., Lu X., Zhao C. (2020). Multifunctional red carbon dots: a theranostic platform for magnetic resonance imaging and fluorescence imaging-guided chemodynamic therapy. Analyst.

[cit11] Wang Y., Li X., Zhao S., Wang B., Song X., Xiao J., Lan M. (2022). Synthesis strategies, luminescence mechanisms, and biomedical applications of near-infrared fluorescent carbon dots. Coord. Chem. Rev..

[cit12] Kozák O., Datta K. K. R., Greplová M., Ranc V., Kašlík J., Zbořil R. (2013). Surfactant-Derived Amphiphilic Carbon Dots with Tunable Photoluminescence. J. Phys. Chem. C.

[cit13] Sun Y.-P., Zhou B., Lin Y., Wang W., Fernando K. A. S., Pathak P., Meziani M. J., Harruff B. A., Wang X., Wang H., Luo P. G., Yang H., Kose M. E., Chen B., Veca L. M., Xie S.-Y. (2006). Quantum-sized carbon dots for bright and colorful photoluminescence. J. Am. Chem. Soc..

[cit14] He H., Liu X., Li S., Wang X., Wang Q., Li J., Wang J., Ren H., Ge B., Wang S., Zhang X., Huang F. (2017). High-Density Super-Resolution Localization Imaging with Blinking Carbon Dots. Anal. Chem..

[cit15] Wang Y., Hu A. (2014). Carbon Quantum Dots: Synthesis, Properties and Applications. J. Mater. Chem. C.

[cit16] Wang Y., Zhu Y., Yu S., Jiang C. (2017). Fluorescent carbon dots: rational synthesis, tunable optical properties and analytical applications. RSC Adv..

[cit17] Ahuja V., Bhatt A. K., Varjani S., Choi K.-Y., Kim S.-H., Yang Y.-H., Bhatia S. K. (2022). Quantum dot synthesis from waste biomass and its applications in energy and bioremediation. Chemosphere.

[cit18] Ðorđević L., Arcudi F., Cacioppo M., Prato M. (2022). A multifunctional chemical toolbox to engineer carbon dots for biomedical and energy applications. Nat. Nanotechnol..

[cit19] Zhi B., Cui Y., Wang S., Frank B. P., Williams D. N., Brown R. P., Melby E. S., Hamers R. J., Rosenzweig Z., Fairbrother D. H., Orr G., Haynes C. L. (2018). Malic Acid Carbon Dots: From Super-resolution Live-Cell Imaging to Highly Efficient Separation. ACS Nano.

[cit20] Zhao Y., Zuo S., Miao M. (2017). The effect of oxygen on the microwave-assisted synthesis of carbon quantum dots from polyethylene glycol. RSC Adv..

[cit21] Zhu H., Wang X., Li Y., Wang Z., Yang F., Yang X. (2009). Microwave synthesis of fluorescent carbon nanoparticles with electrochemiluminescence properties. Chem. Commun..

[cit22] Ergüder Ö., Keskin S. Ş., Nar I., Trabzon L., Ünlü C. (2022). Aflatoxin B1 Acts as an Effective Energy Donor to Enhance Fluorescence of Yellow Emissive Carbon Dots. ACS Omega.

[cit23] Liu H., He Z., Jiang L.-P., Zhu J.-J. (2015). Microwave-assisted synthesis of wavelength-tunable photoluminescent carbon nanodots and their potential applications. ACS Appl. Mater. Interfaces.

[cit24] Cao M., Li Y., Zhao Y., Shen C., Zhang H., Huang Y. (2019). A novel method for the preparation of solvent-free, microwave-assisted and nitrogen-doped carbon dots as fluorescent probes for chromium(vi) detection and bioimaging. RSC Adv..

[cit25] Zhai X., Zhang P., Liu C., Bai T., Li W., Dai L., Liu W. (2012). Highly luminescent carbon nanodots by microwave-assisted pyrolysis. Chem. Commun..

[cit26] Cui J., Zhu X., Liu Y., Liang L., Peng Y., Wu S., Zhao Y. (2022). N-Doped Carbon Dots as Fluorescent “Turn-Off” Nanosensors for Ascorbic Acid and Fe^3+^ Detection. ACS Appl. Nano Mater..

[cit27] Murali G., Kwon B., Kang H., Modigunta J. K. R., Park S., Lee S., Lee H., Park Y. H., Kim J., Park S. Y., Kim Y.-J., In I. (2022). Hematoporphyrin Photosensitizer-Linked Carbon Quantum Dots for Photodynamic Therapy of Cancer Cells. ACS Appl. Nano Mater..

[cit28] Hashemzadeh I., Hasanzadeh A., Radmanesh F., Chegeni B. K., Hosseini E. S., Kiani J., Shahbazi A., Naseri M., Fatahi Y., Nourizadeh H., Azar B. K. Y., Aref A. R., Liu Y., Hamblin M. R., Karimi M. (2021). Polyethylenimine-Functionalized Carbon Dots for Delivery of CRISPR/Cas9 Complexes. ACS Appl. Bio Mater..

[cit29] Mondal S., Yucknovsky A., Akulov K., Ghorai N., Schwartz T., Ghosh H. N., Amdursky N. (2019). Efficient Photosensitizing Capabilities and Ultrafast Carrier Dynamics of Doped Carbon Dots. J. Am. Chem. Soc..

[cit30] Singaravelu C. M., Deschanels X., Rey C., Causse J. (2021). Solid-State Fluorescent Carbon Dots for Fluorimetric Sensing of Hg^2+^. ACS Appl. Nano Mater..

[cit31] Purbia R., Paria S. (2016). A simple turn on fluorescent sensor for the selective detection of thiamine using coconut water derived luminescent carbon dots. Biosens. Bioelectron..

[cit32] Gómez I. J., Sulleiro M. V., Dolečková A., Pizúrová N., Medalová J., Roy R., Nečas D., Zajíčková L. (2021). Exploring the Emission Pathways in Nitrogen-Doped Graphene Quantum Dots for Bioimaging. J. Phys. Chem. C.

[cit33] Wu Q., Wang L., Yan Y., Li S., Yu S., Wang J., Huang L. (2022). Chitosan-Derived Carbon Dots with Room-Temperature Phosphorescence and Energy Storage Enhancement Properties. ACS Sustainable Chem. Eng..

[cit34] Wang Q., Zheng H., Long Y., Zhang L., Gao M., Bai W. (2011). Microwave-hydrothermal synthesis of fluorescent carbon dots from graphite oxide. Carbon.

[cit35] Ding H., Wei J.-S., Zhong N., Gao Q.-Y., Xiong H.-M. (2017). Highly Efficient Red-Emitting Carbon Dots with Gram-Scale Yield for Bioimaging. Langmuir.

[cit36] Tang L., Ji R., Cao X., Lin J., Jiang H., Li X., Teng K. S., Luk C. M., Zeng S., Hao J., Lau S. P. (2012). Deep ultraviolet photoluminescence of water-soluble self-passivated graphene quantum dots. ACS Nano.

[cit37] Saravanan A., Maruthapandi M., Das P., Ganguly S., Margel S., Luong J. H. T., Gedanken A. (2020). Applications of N-Doped Carbon Dots as Antimicrobial Agents, Antibiotic Carriers, and Selective Fluorescent Probes for Nitro Explosives. ACS Appl. Bio Mater..

[cit38] Zhuo Y., Prestat E., Kinloch I. A., Bissett M. A. (2022). Self-Assembled 1T-MoS_2_/Functionalized Graphene Composite Electrodes for Supercapacitor Devices. ACS Appl. Energy Mater..

[cit39] Ismaili H., Geng D., Sun A. X., Kantzas T. T., Workentin M. S. (2011). Light-Activated Covalent Formation of Gold Nanoparticle-Graphene and Gold Nanoparticle-Glass Composites. Langmuir.

[cit40] Meragawi S. E., Akbari A., Hernandez S., Tanksale A., Majumder M. (2020). Efficient Permeance Recovery of Organically Fouled Graphene Oxide Membranes. ACS Appl. Bio Mater..

[cit41] Stobinski L., Lesiak B., Malolepszy A., Mazurkiewicz M., Mierzwa B., Zemek J., Jiricek P., Bieloshapka I. (2014). Graphene oxide and reduced graphene oxide studied by the XRD, TEM and electron spectroscopy methods. J. Electron Spectrosc. Relat. Phenom..

[cit42] Tang F., Wang C., Wang J., Wang X., Li L. (2014). Fluorescent Organic Nanoparticles with Enhanced Fluorescence by Self-Aggregation and their Application to Cellular Imaging. ACS Appl. Mater. Interfaces.

[cit43] Li S., He J., Xu Q.-H. (2020). Aggregation of Metal-Nanoparticle-Induced Fluorescence Enhancement and Its Application in Sensing. ACS Omega.

