# Supplementary material for: Improved citric acid-derived carbon dots synthesis through microwave-based heating in a hydrothermal pressure vessel
Source: RSC Adv. 2022 Nov 11;12(50):32401–14. doi: 10.1039/d2ra06420k (PMC9651130; doi:10.1039/d2ra06420k)
Supplement: RA-012-D2RA06420K-s001 [file RA-012-D2RA06420K-s001.pdf]

Electronic Supplementary Material (ESI) for RSC Advances.
This journal is © The Royal Society of Chemistry 2022

Improved Citric Acid-Derived Carbon Dots Synthesis Through Microwave-Based Heating in Hydrothermal Pressure Vessel

Jorns, M.; Strickland, S.; Mullins, M; Pappas, D.*

Supporting Information

Section A – TEM Images of CDs Samples in PBS

Figure S1. Representative TEM images of oven CDs resuspended in PBS. (A-E)

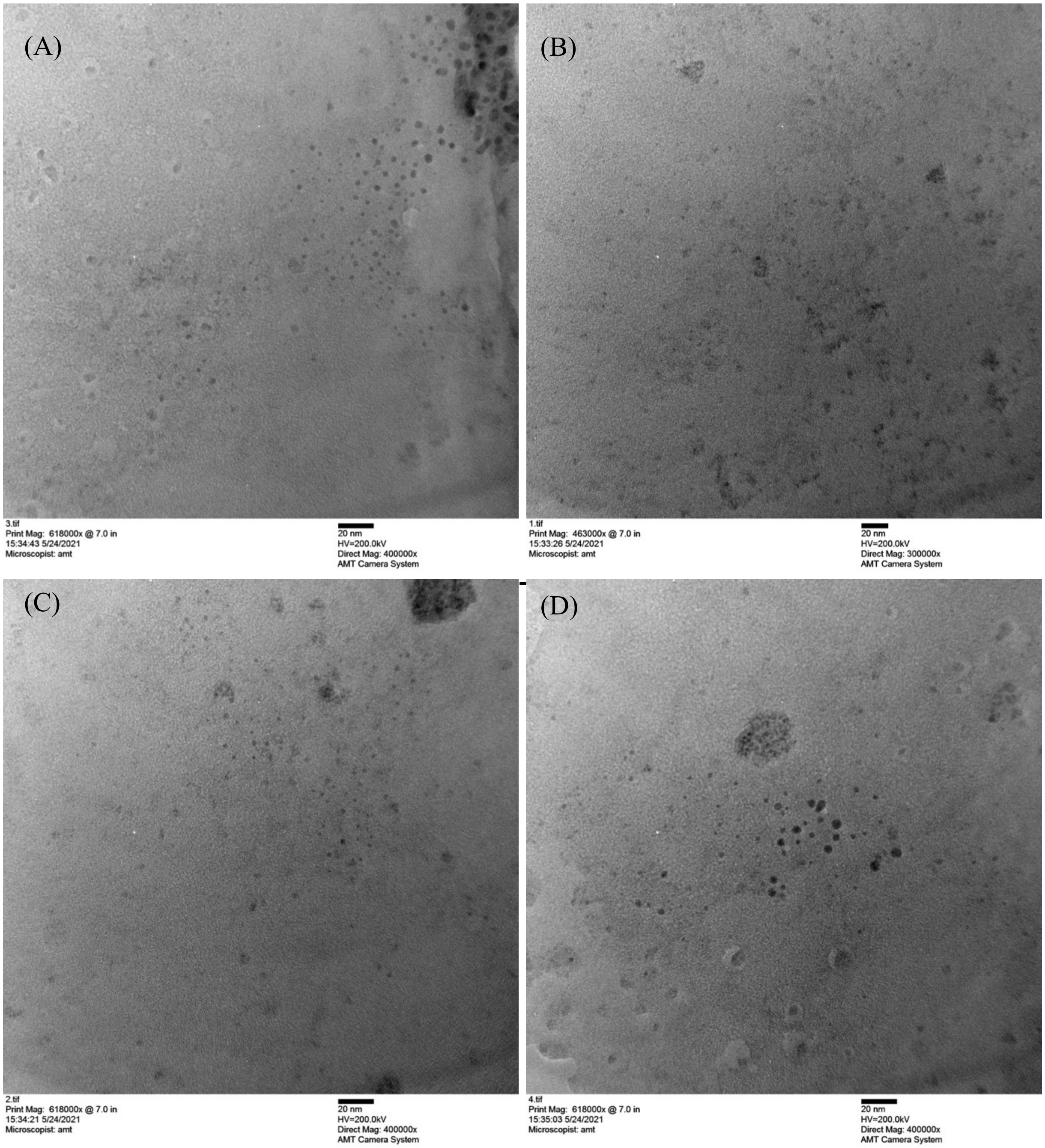

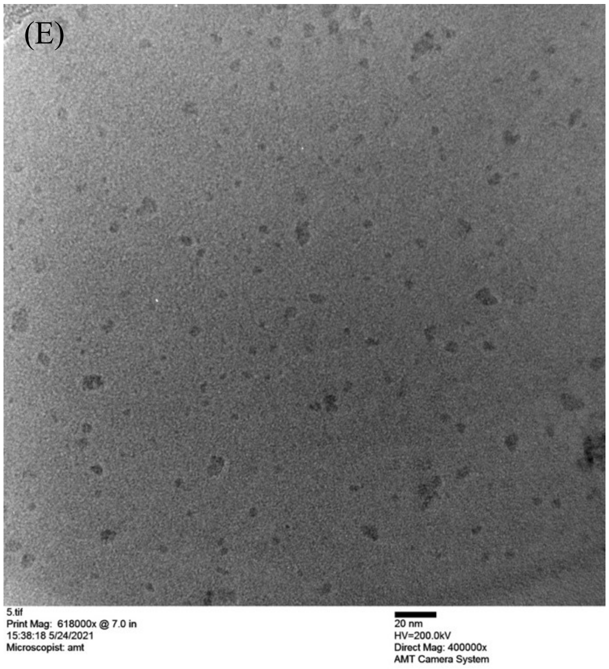

Figure S2. Representative TEM images of microwave CDs heated for 40 seconds and resuspended in PBS. (A-E)

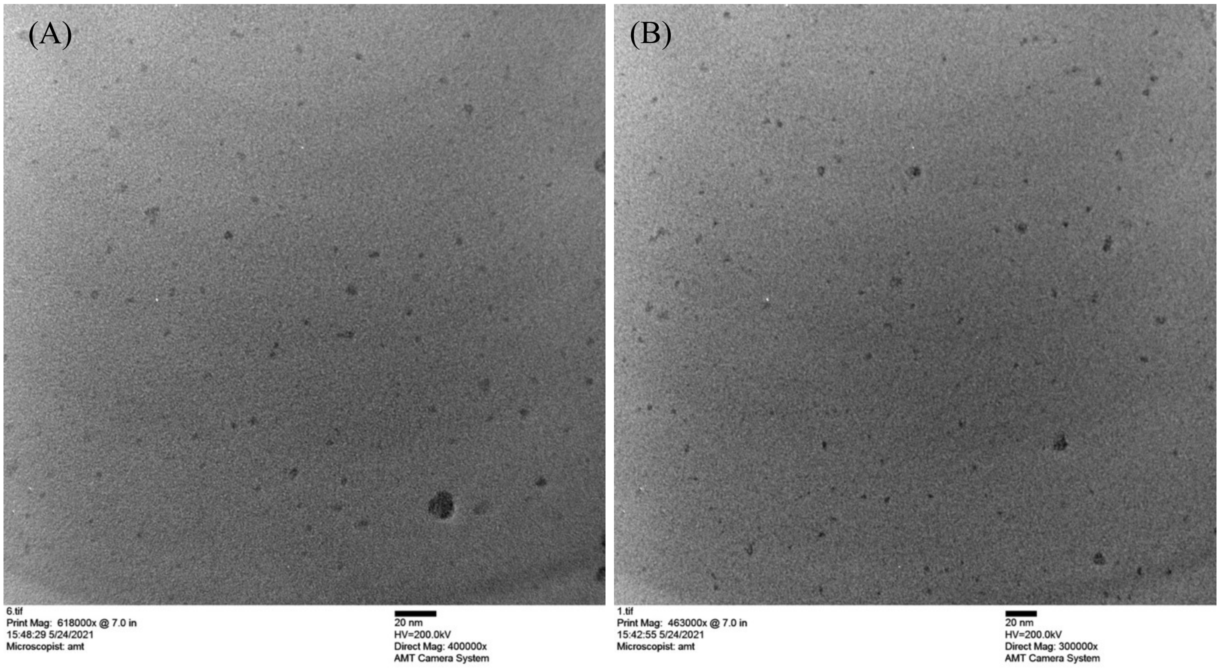

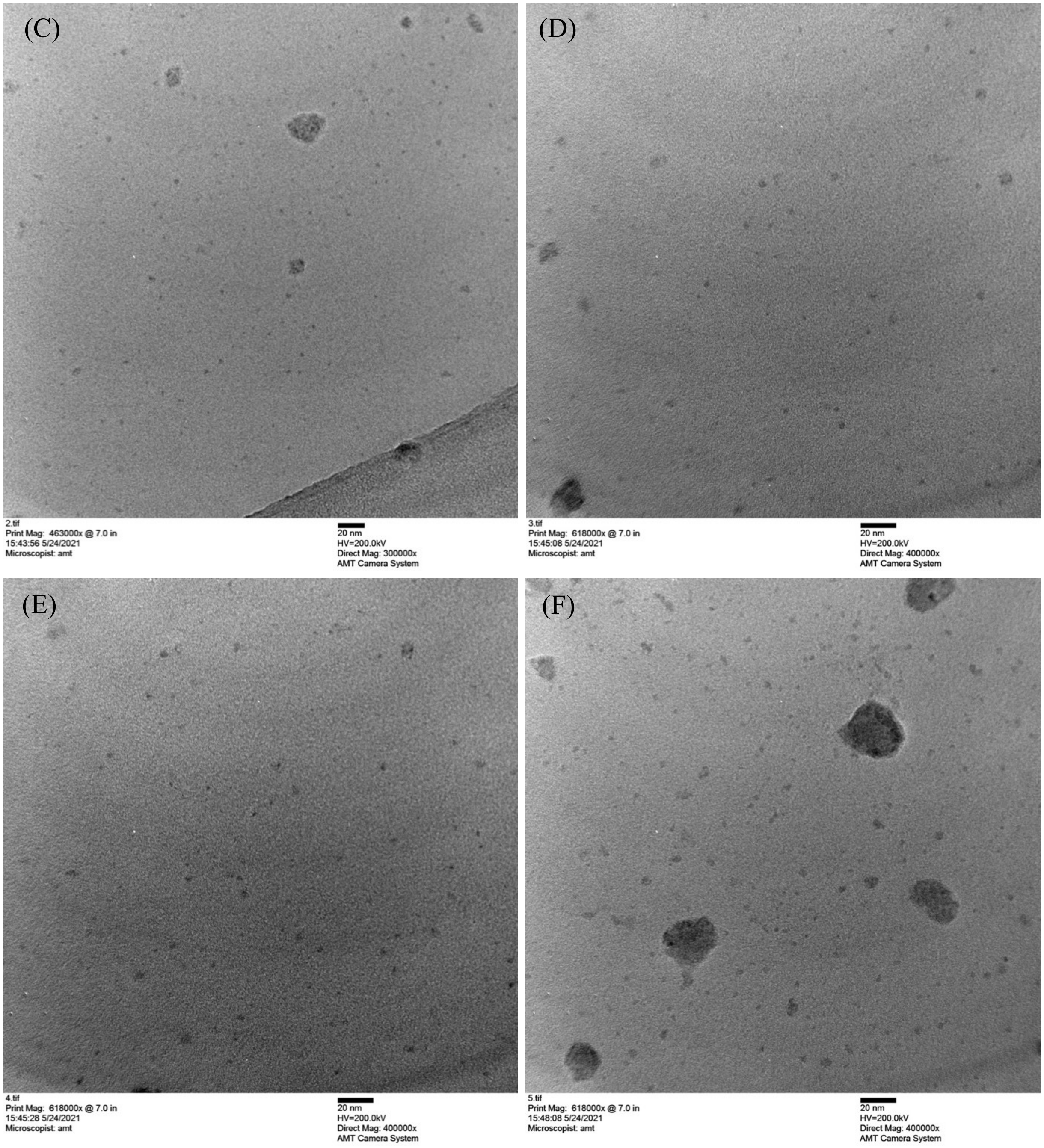

Figure S3. Representative TEM images of microwave CDs heated for 60 seconds and resuspended in PBS. (A-J)

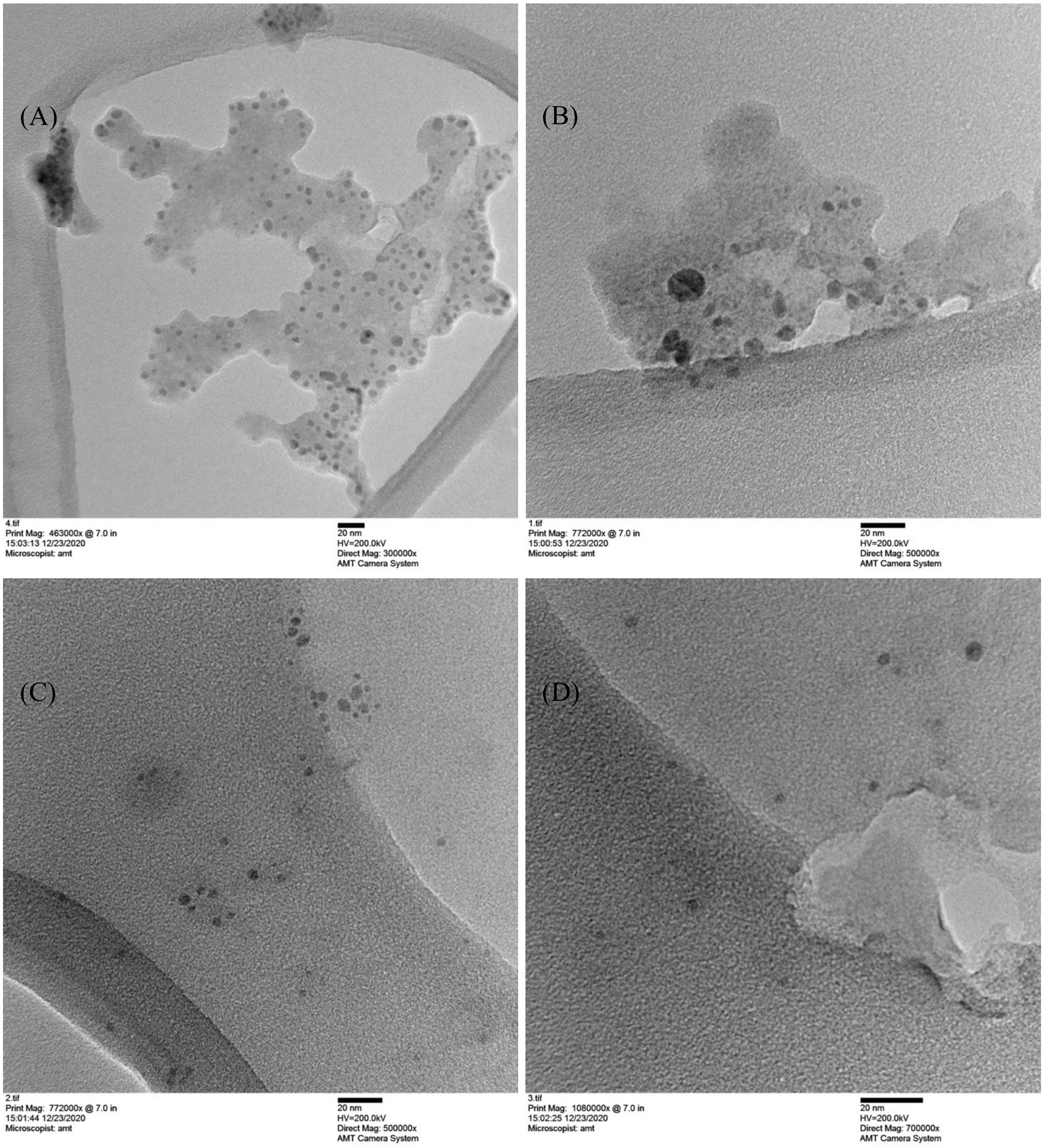
(A)
4.tif
Print Mag: 463000x @ 7.0 in
15:03:13 12/23/2020
Microscopist: amt
20 nm
HV=200.0kV
Direct Mag: 300000x
AMT Camera System
(B)
1.tif
Print Mag: 772000x @ 7.0 in
15:00:53 12/23/2020
Microscopist: amt
20 nm
HV=200.0kV
Direct Mag: 500000x
AMT Camera System
(C)
2.tif
Print Mag: 772000x @ 7.0 in
15:01:44 12/23/2020
Microscopist: amt
20 nm
HV=200.0kV
Direct Mag: 500000x
AMT Camera System
(D)
3.tif
Print Mag: 1080000x @ 7.0 in
15:02:25 12/23/2020
Microscopist: amt
20 nm
HV=200.0kV
Direct Mag: 700000x
AMT Camera System

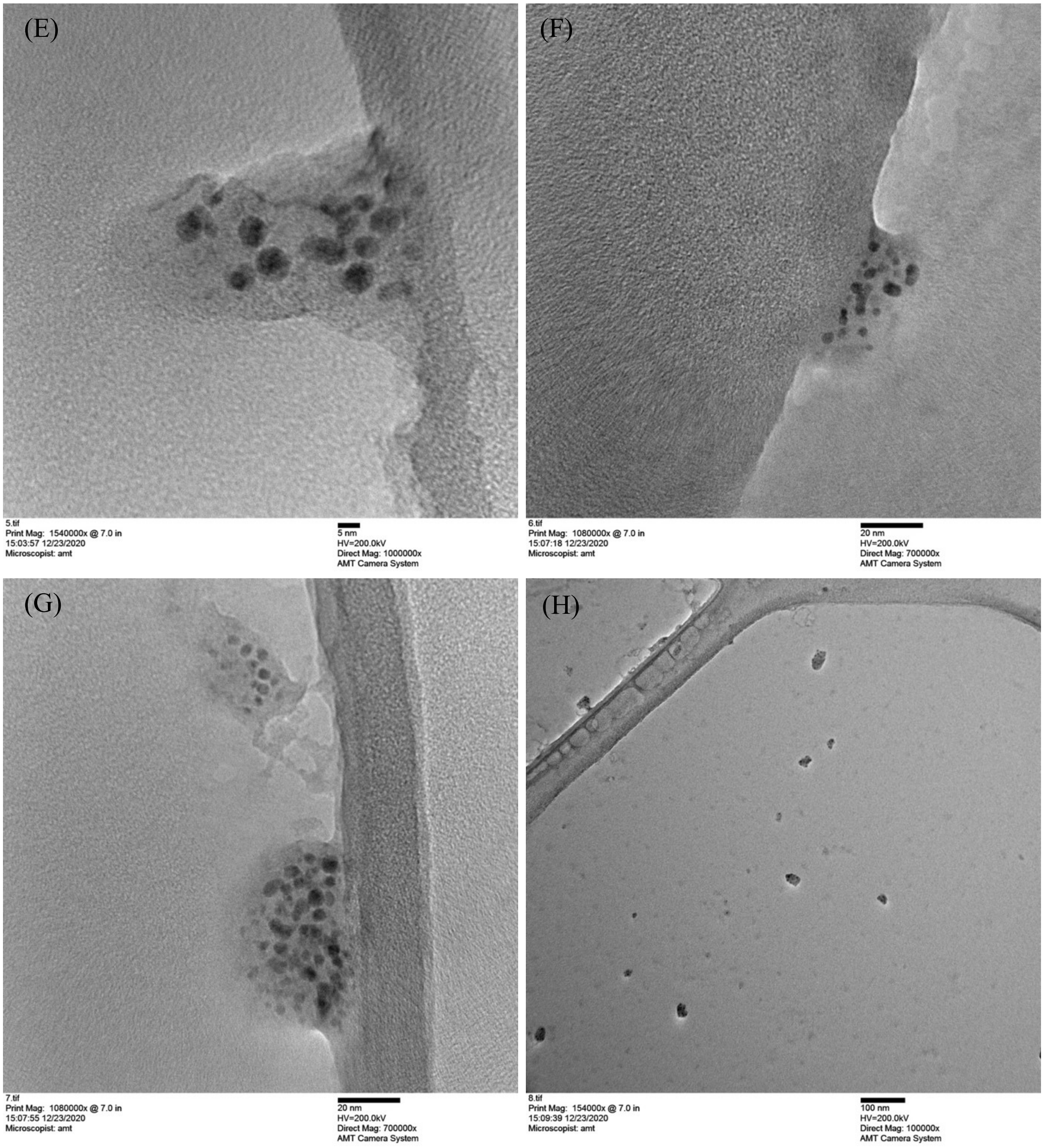
(E)
5.tif
Print Mag: 1540000x @ 7.0 in
15:03:57 12/23/2020
Microscopist: amt
5 nm
HV=200.0kV
Direct Mag: 1000000x
AMT Camera System
(F)
6.tif
Print Mag: 1080000x @ 7.0 in
15:07:18 12/23/2020
Microscopist: amt
20 nm
HV=200.0kV
Direct Mag: 700000x
AMT Camera System
(G)
7.tif
Print Mag: 1080000x @ 7.0 in
15:07:55 12/23/2020
Microscopist: amt
20 nm
HV=200.0kV
Direct Mag: 700000x
AMT Camera System
(H)
8.tif
Print Mag: 154000x @ 7.0 in
15:09:39 12/23/2020
Microscopist: amt
100 nm
HV=200.0kV
Direct Mag: 100000x
AMT Camera System

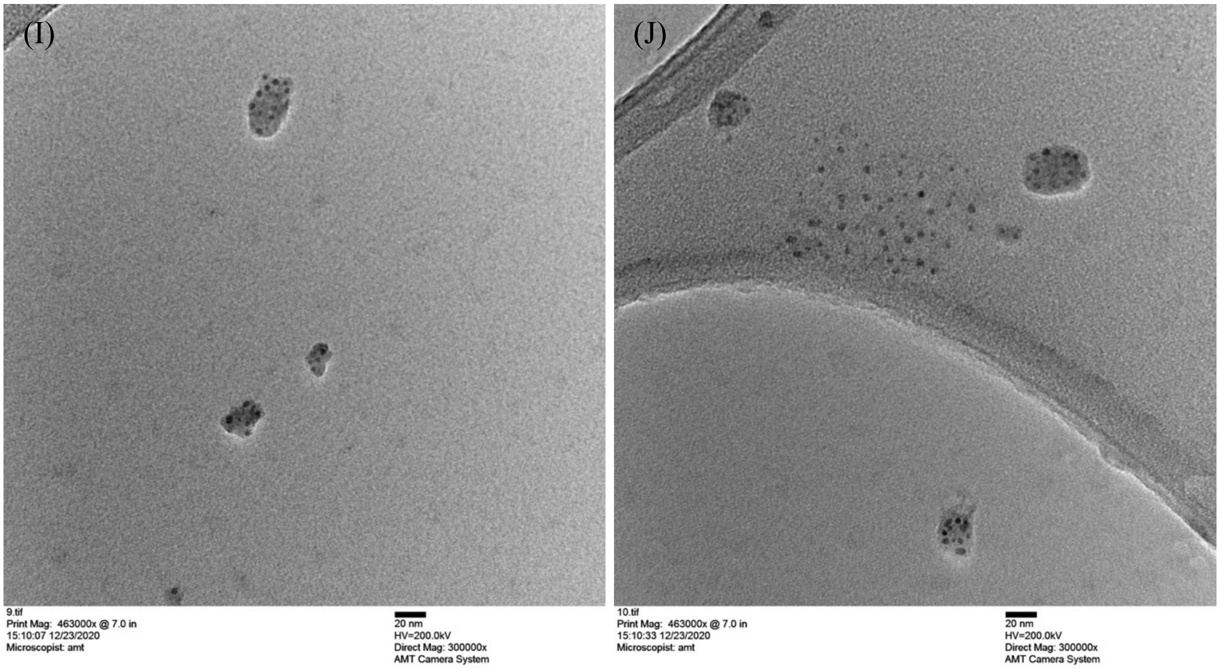

Figure S4. Representative TEM images of microwave CDs heated for 80 seconds and resuspended in PBS. (A-F)

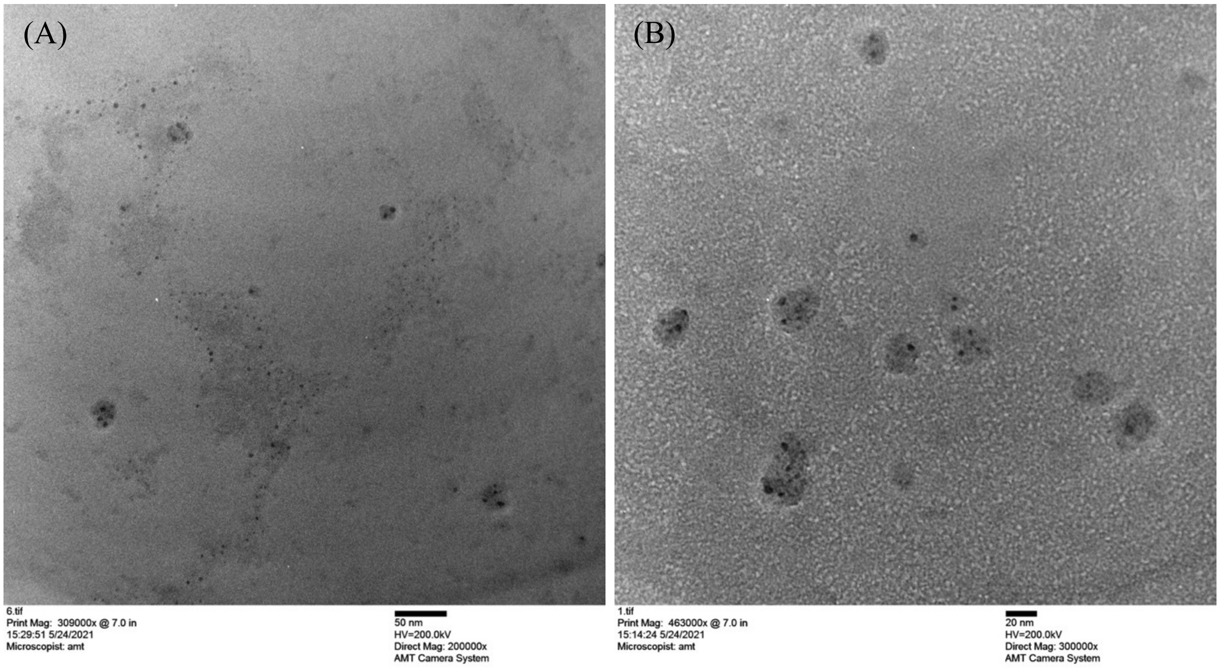

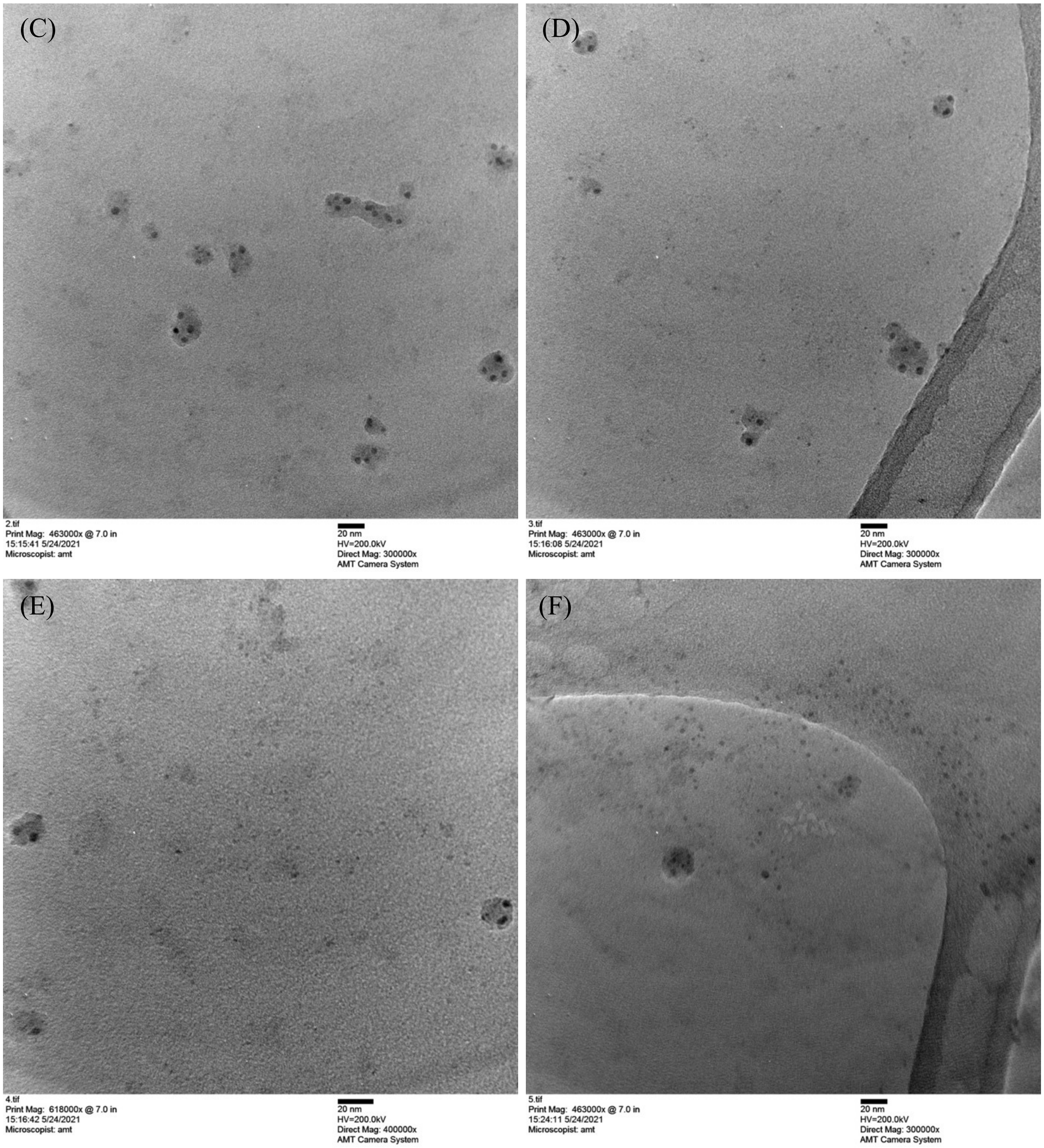

Figure S5. Representative TEM images of microwave CDs heated for 100 seconds and resuspended in PBS. (A-F)

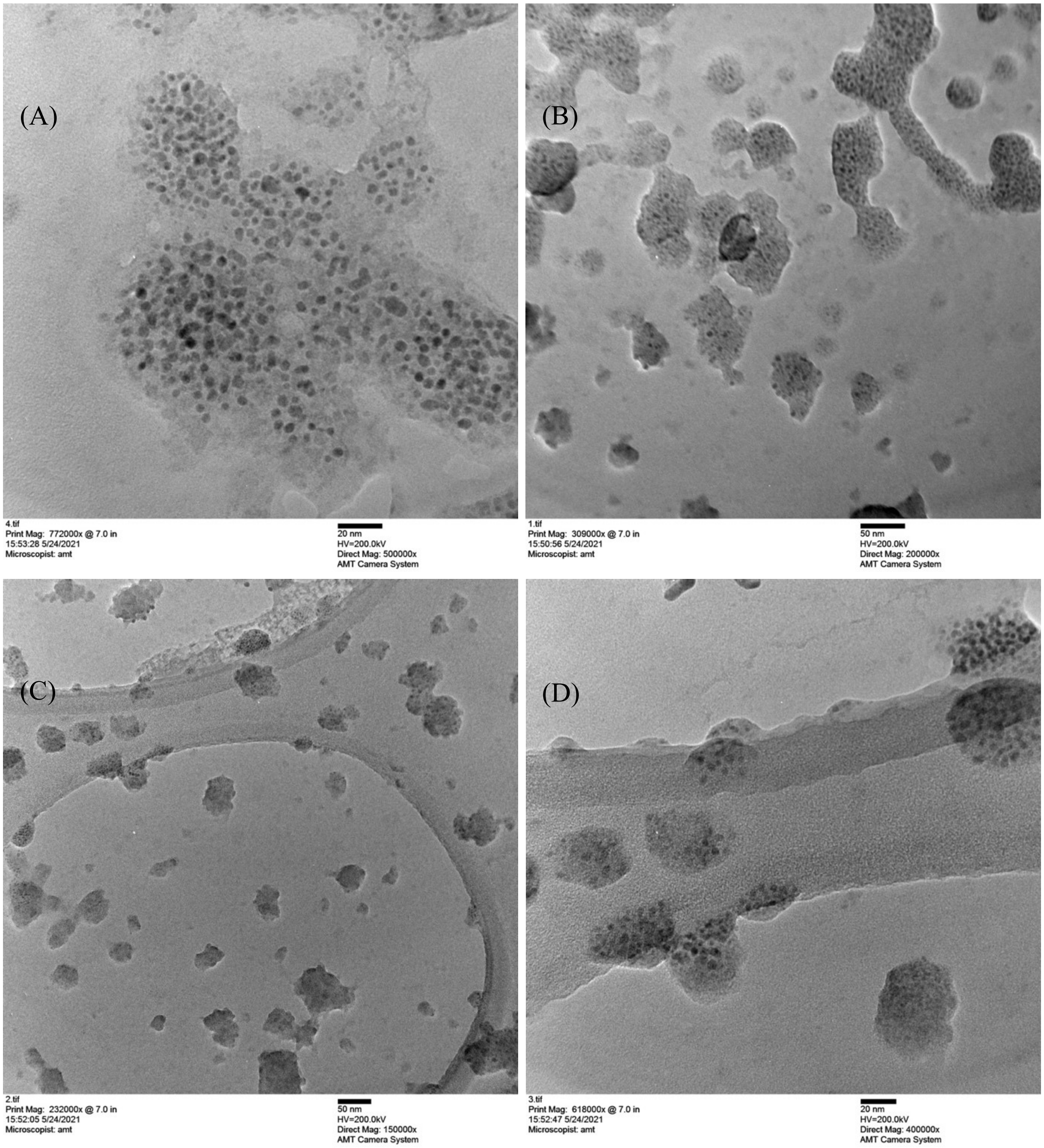
(A)
4.tif
Print Mag: 772000x @ 7.0 in
15:53:28 5/24/2021
Microscopist: amt
20 nm
HV=200.0kV
Direct Mag: 500000x
AMT Camera System
(B)
1.tif
Print Mag: 309000x @ 7.0 in
15:50:56 5/24/2021
Microscopist: amt
50 nm
HV=200.0kV
Direct Mag: 200000x
AMT Camera System
(C)
2.tif
Print Mag: 232000x @ 7.0 in
15:52:05 5/24/2021
Microscopist: amt
50 nm
HV=200.0kV
Direct Mag: 150000x
AMT Camera System
(D)
3.tif
Print Mag: 618000x @ 7.0 in
15:52:47 5/24/2021
Microscopist: amt
20 nm
HV=200.0kV
Direct Mag: 400000x
AMT Camera System

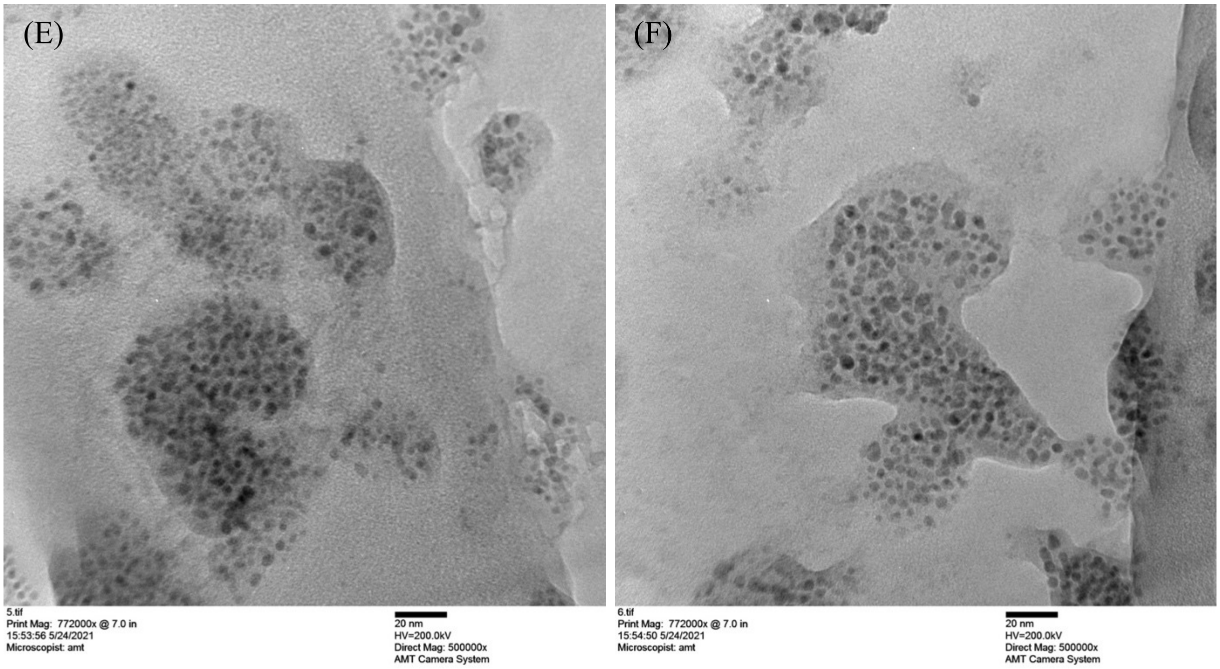

<u>Section B – UV-Vis Absorption Measurements for Standard Addition Analysis</u>

The relative particle concentration of CDs samples heated in the oven and the various times in the microwave was determined using the standard addition method. Each CDs solution (suspended in DI water) was sufficiently diluted to accurately measure initial absorbance intensities, then spiked with 4 aliquots of 30 μL of undiluted CDs solution while taking absorbance measurements after each addition (Figures 6, 8, 10, 12, and 14). The reported dilutions in these plots for each sample refer to the percentage of the original CDs solution per total volume. Calibration curves were plotted of absorbance versus volume of aliquot added to generate a linear trendline (Figures 7, 9, 11, 13, and 15). The x-intercept was then calculated from this formula as the relative estimation of particle concentration per microliter of diluted sample since each volume of aliquot would be theoretically supplying a consistent unknown concentration of CDs. This value was then multiplied by the dilution factor to determine relative density of undiluted sample. These particle concentrations only serve to compare CDs samples and do not provide actual counts of particles per volume.

Figure S6.

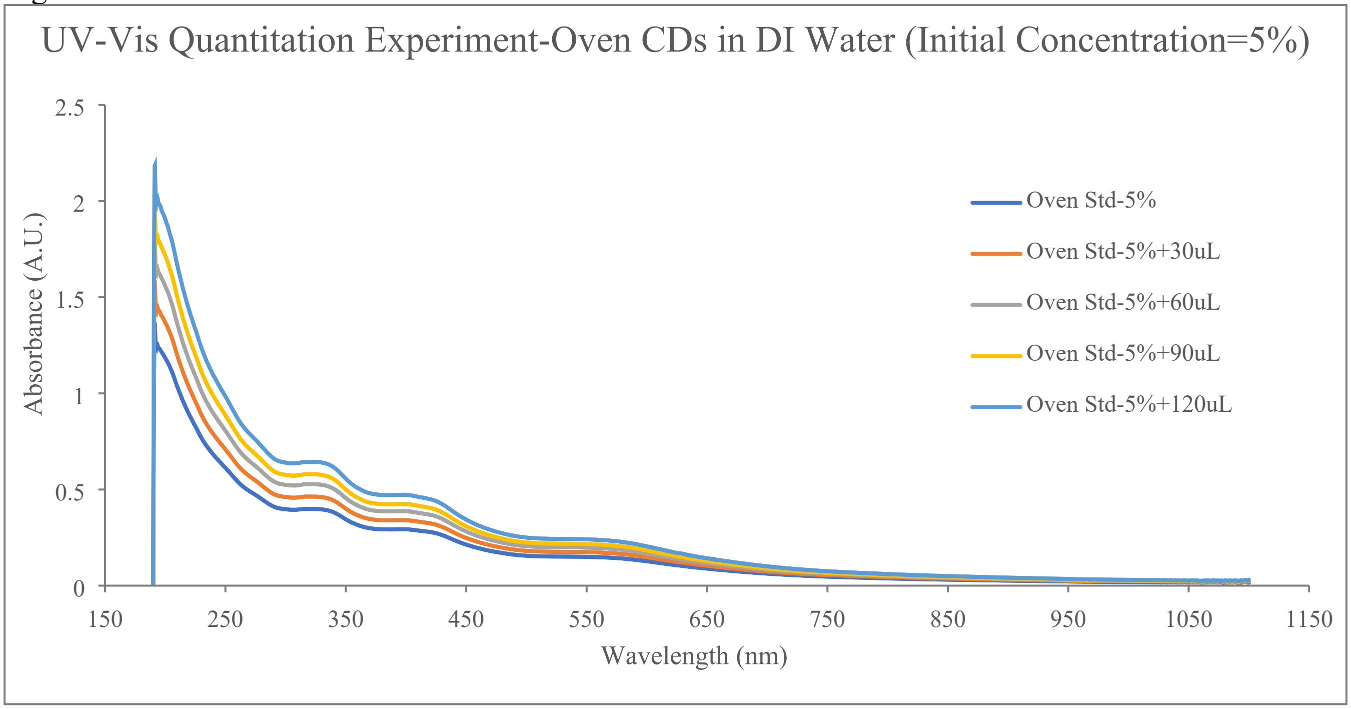

Figure S7.

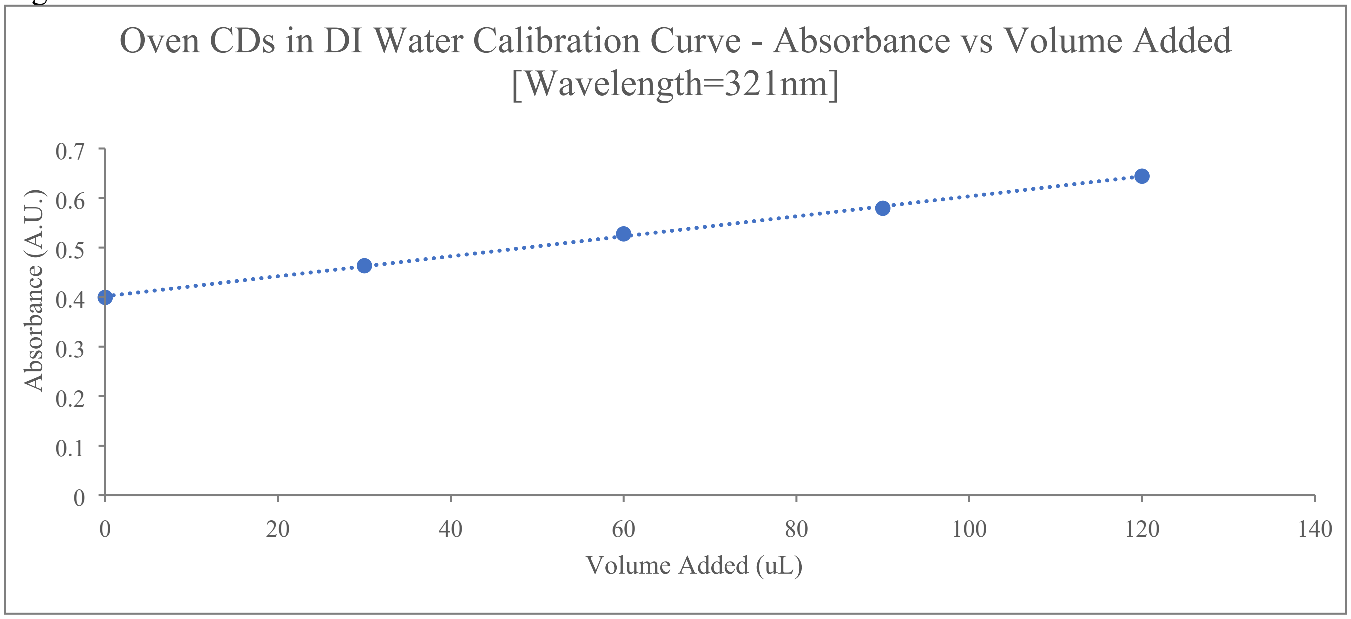

x-intercept = 200.85 units/µL
Original relative sample concentration (Oven CDs) = **4017 units/µL**

Figure S8.

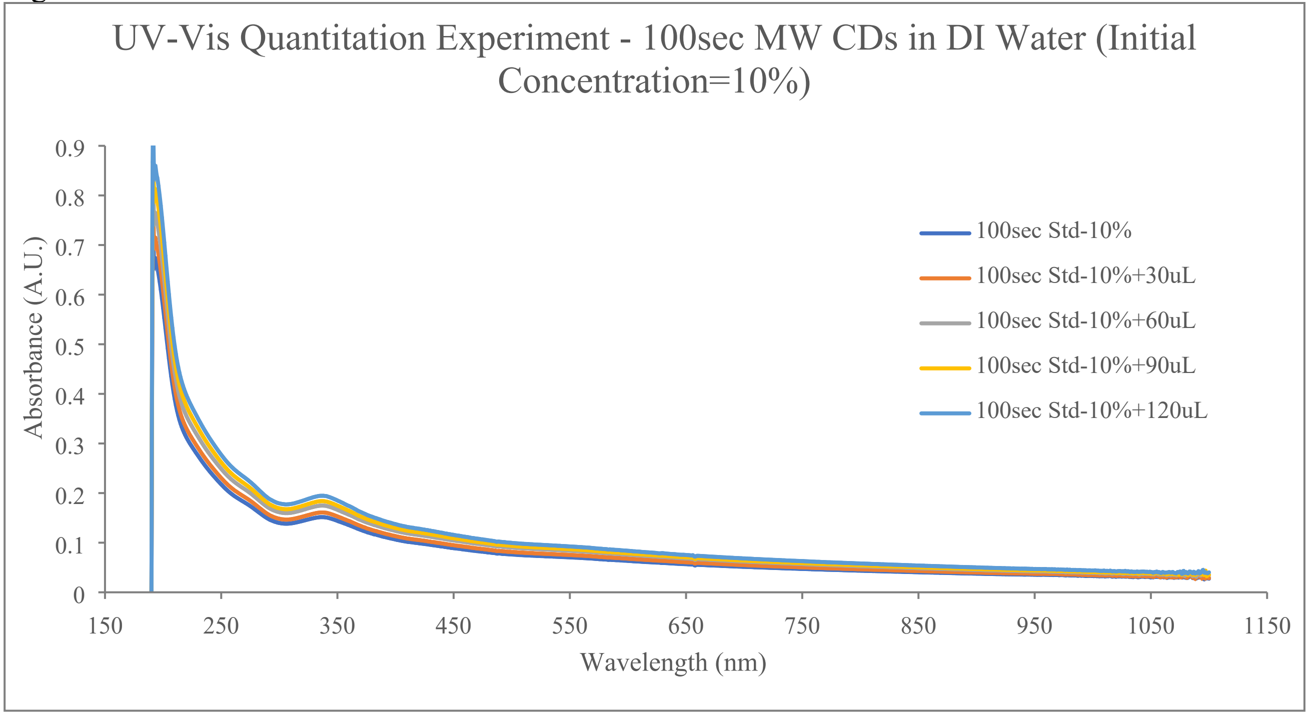

Figure S9.

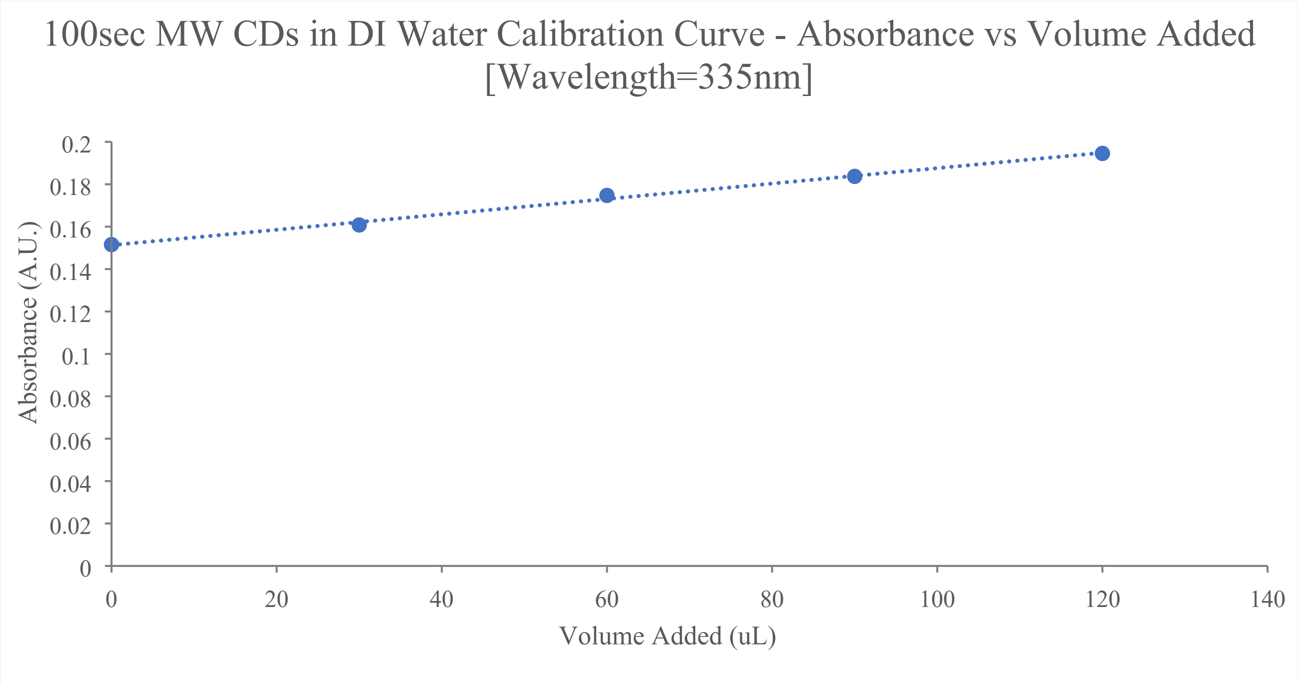

x-intercept = 378.25 units/µL
Original relative sample concentration (100sec in MW CDs) = **3782.5 units/µL**

Figure S10.

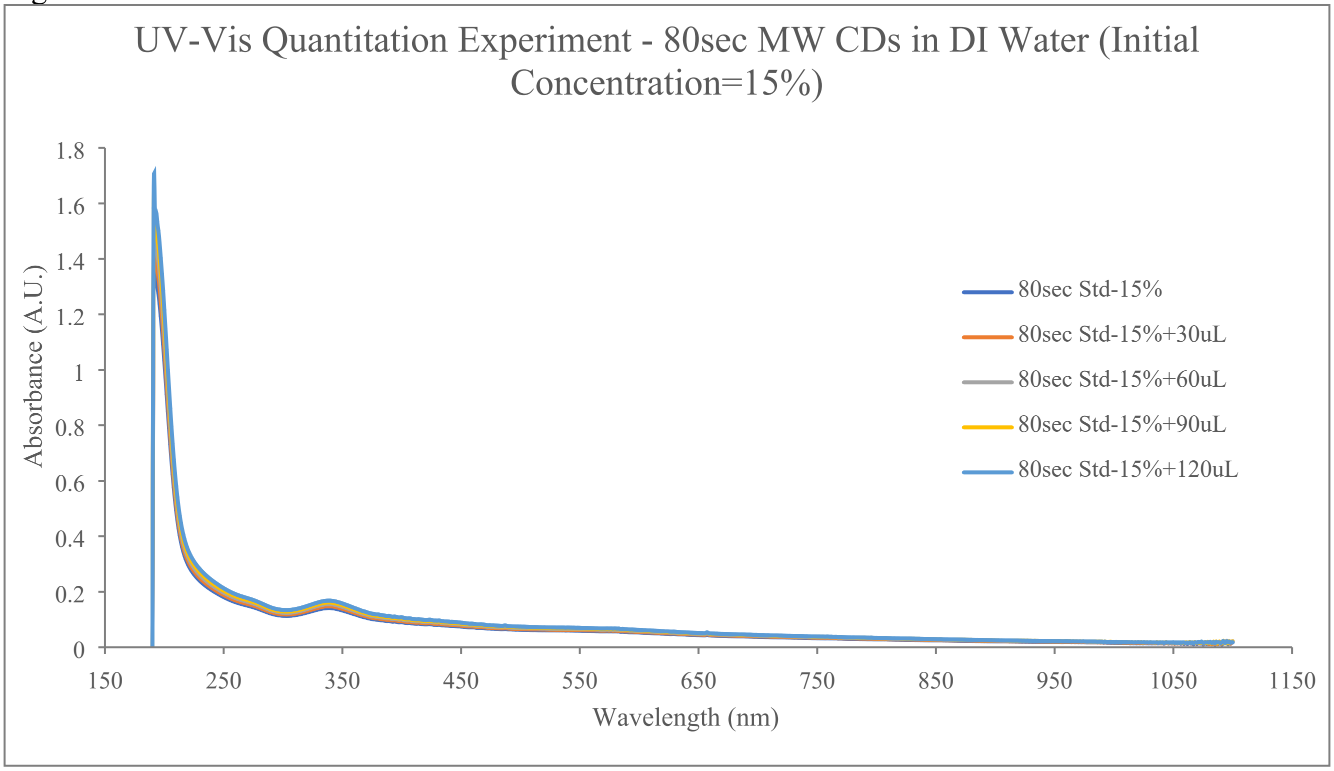

Figure S11.

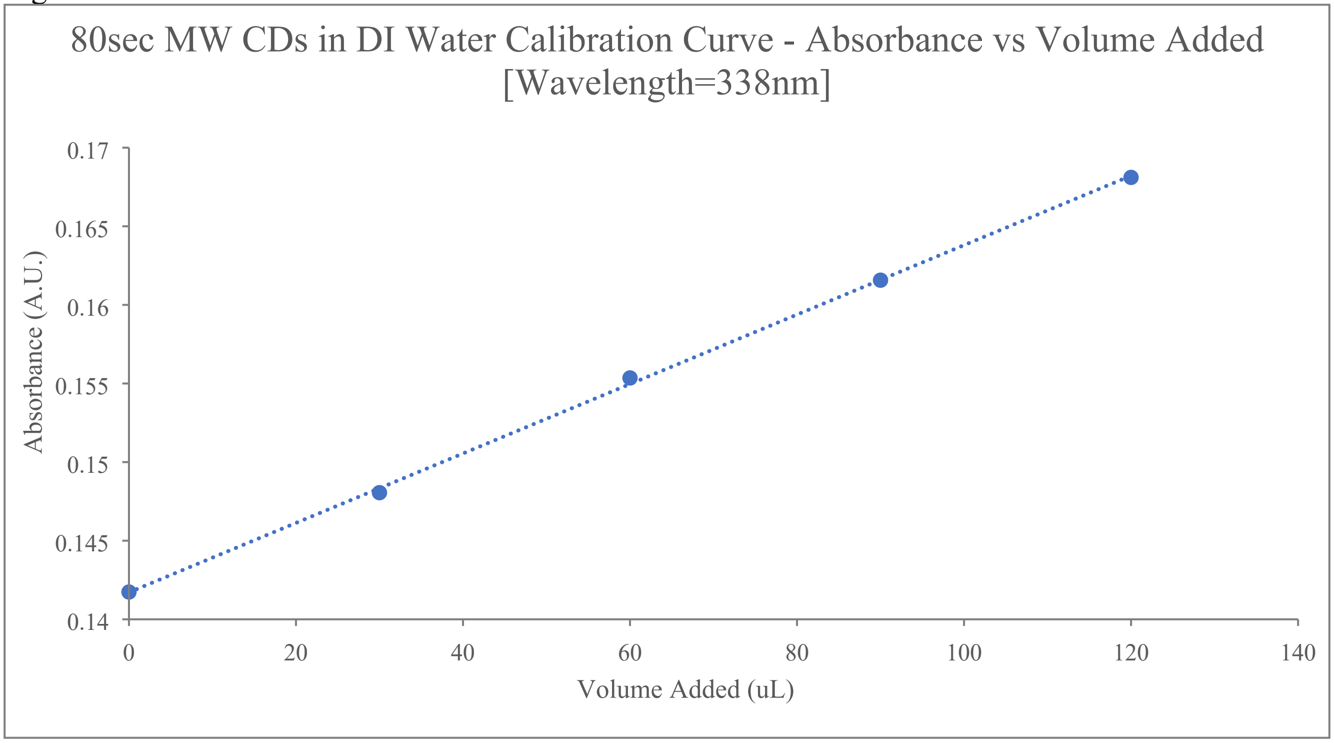

x-intercept = 708.5 units/µL

Original relative sample concentration (80sec in MW) = **4723.3 units/µL**

Figure S12.

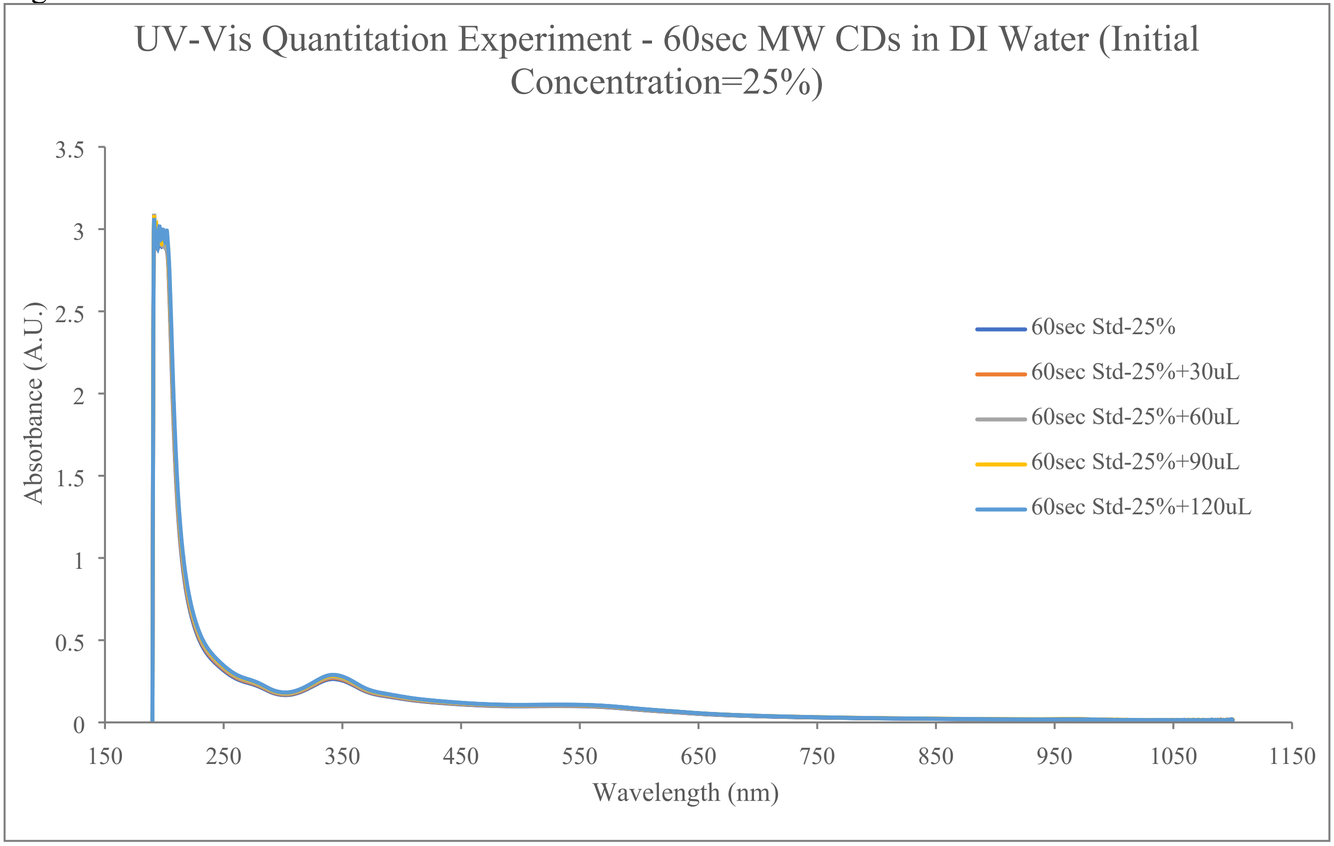

Figure S13.

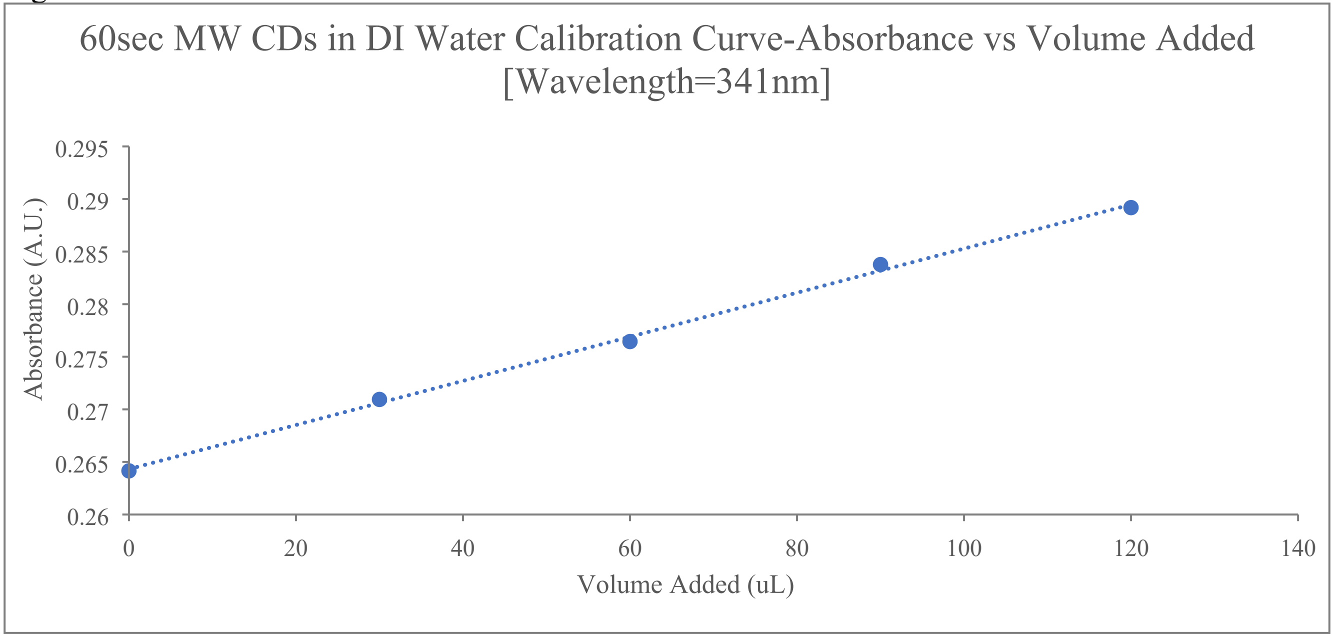

x-intercept = 1321.5 units/µL
Original relative sample concentration (60sec in MW) = **5286 units/µL**

Figure S14.

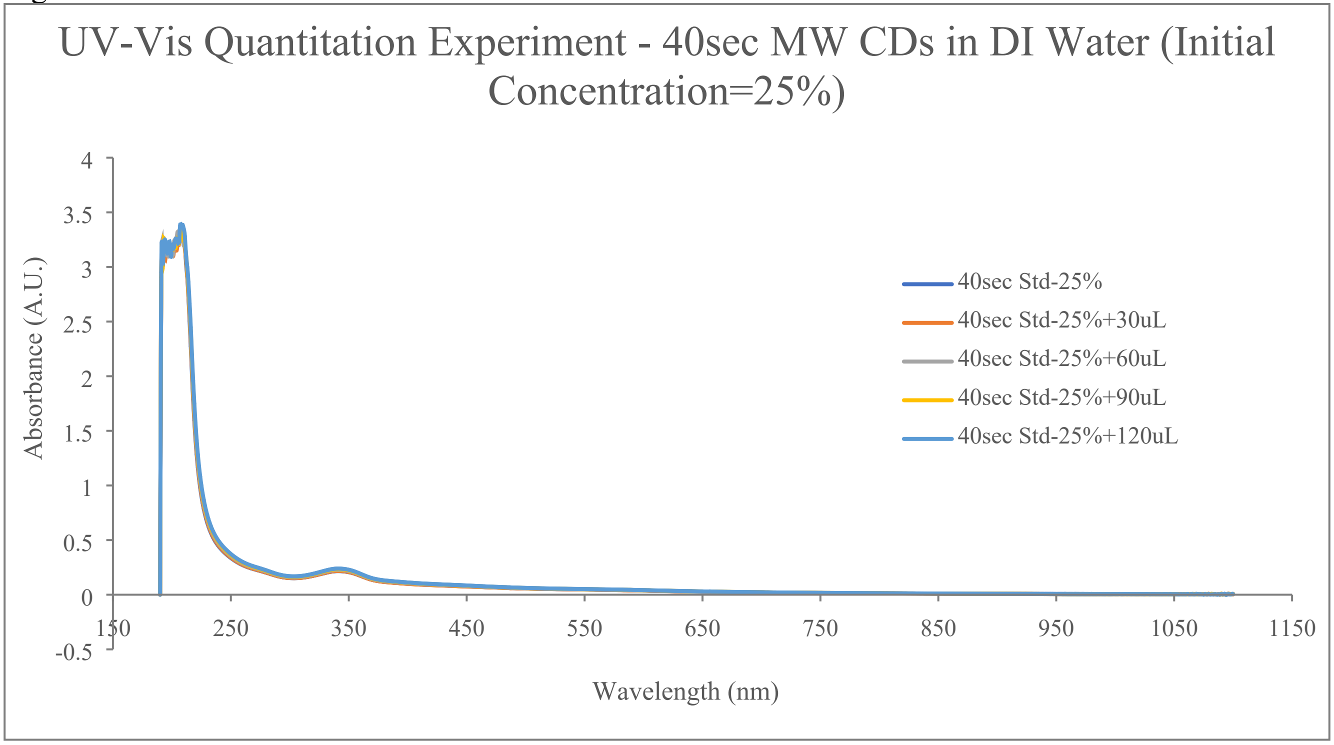

Figure S15.

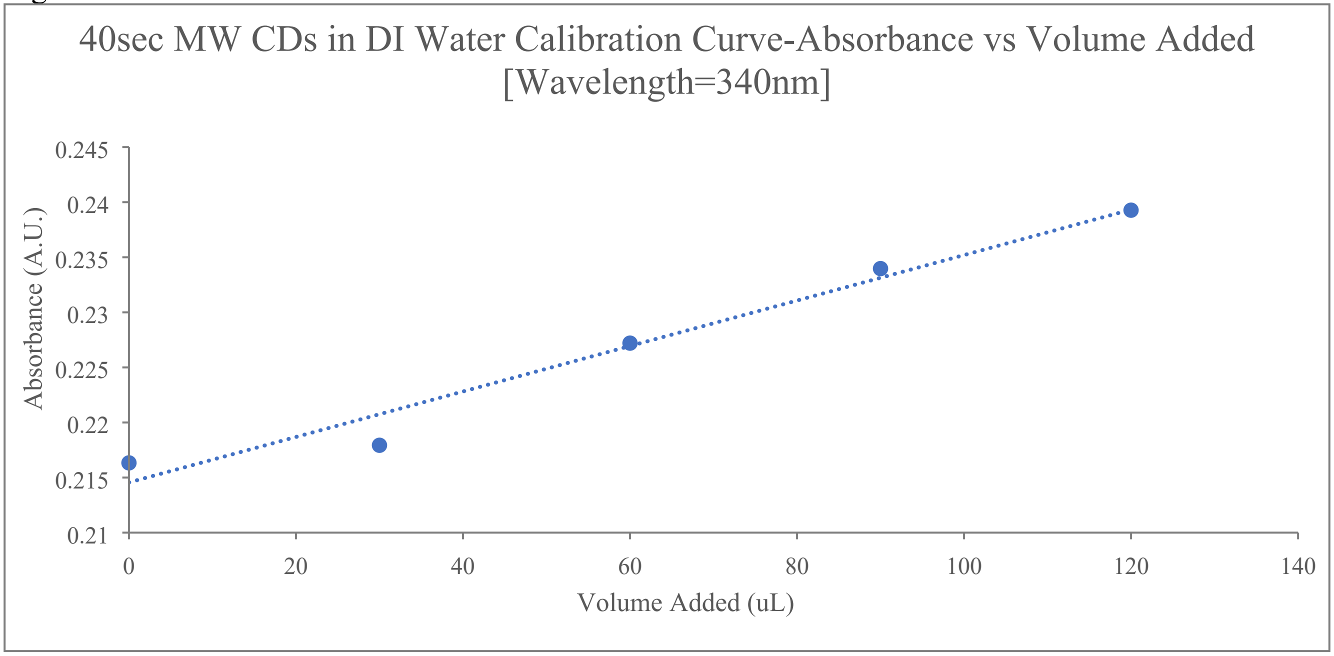

x-intercept = 1073 units/µL
Original relative sample concentration (40sec in MW) = **4292 units/µL**

Section C – SEM/EDS data

Solid CDs samples (oven CDs and 100 second MW CDs) were imaged and assessed by scanning electron microscopy (SEM) and energy dispersive X-ray spectroscopy (EDS). The samples were each imaged at three distinct locations to obtain a more comprehensive visualization of the product and determine average elemental profiles.

Oven CDs:

Figure S16. Oven CDs SEM images. (A) Site #1, (B) Site #2, and (C) Site #3.

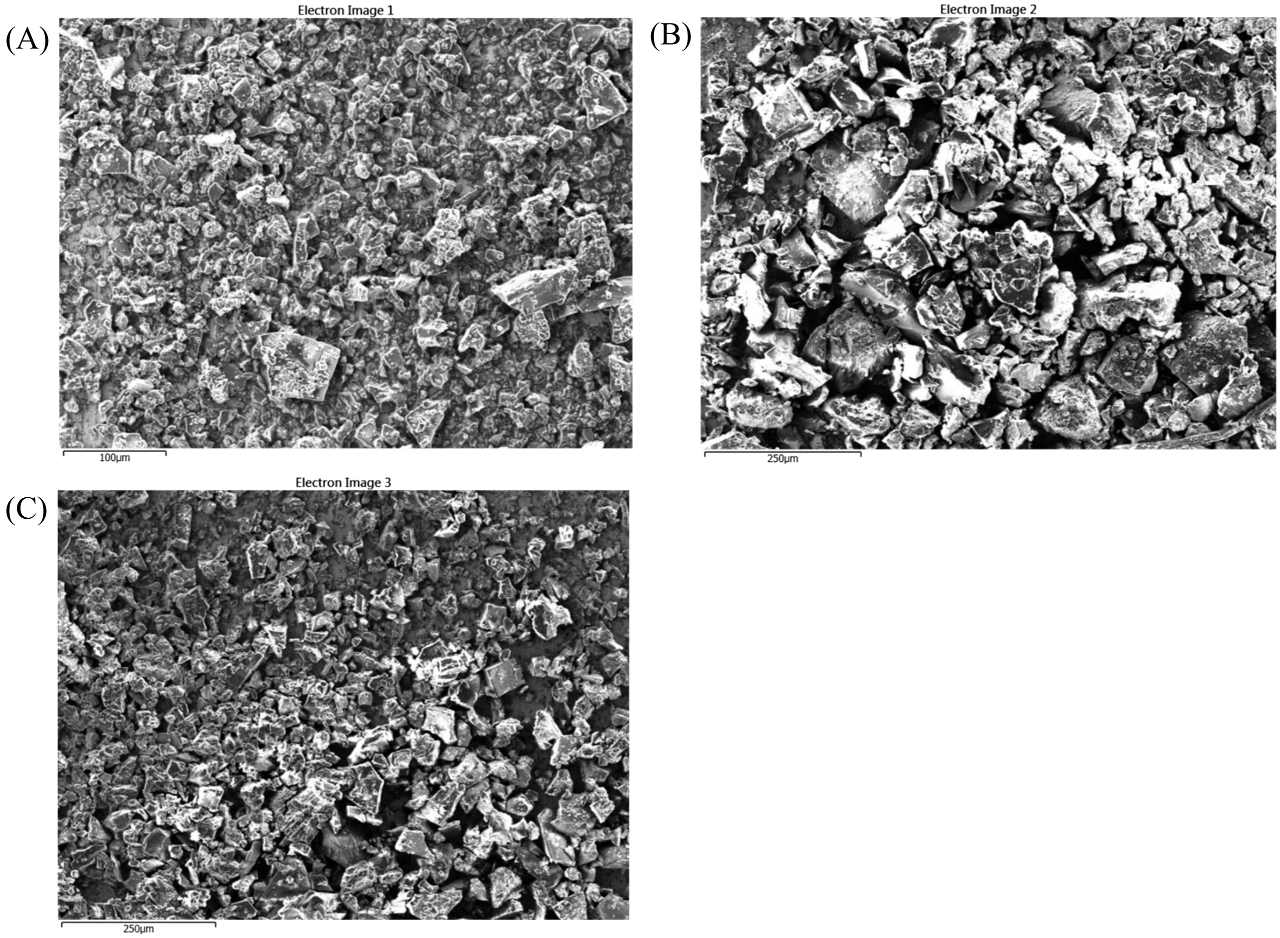

Table S1.

Element	Site 1 (% ± σ)	Site 2 (% ± σ)	Site 3 (% ± σ)	Average (± STD)
C	43.6% ± 0.1	48.1% ± 0.2	47.2% ± 0.2	46.3% ± 0.3
N	25.2% ± 0.2	27.4% ± 0.3	26.6% ± 0.2	26.4% ± 0.4
O	31.2% ± 0.1	24.4% ± 0.2	25.8% ± 0.1	27.1% ± 0.2
Ca	---------------	0.2% ± 0.0	0.4% ± 0.0	0.2% ± 0.0

Oven EDS data. Calcium is considered as a contaminant and not an additional dopant since it was not present in the starting materials.

100sec MW CDs:

Figure S17. 100sec MW CDs SEM images. (A) Site #1, (B) Site #2, and (C) Site #3.

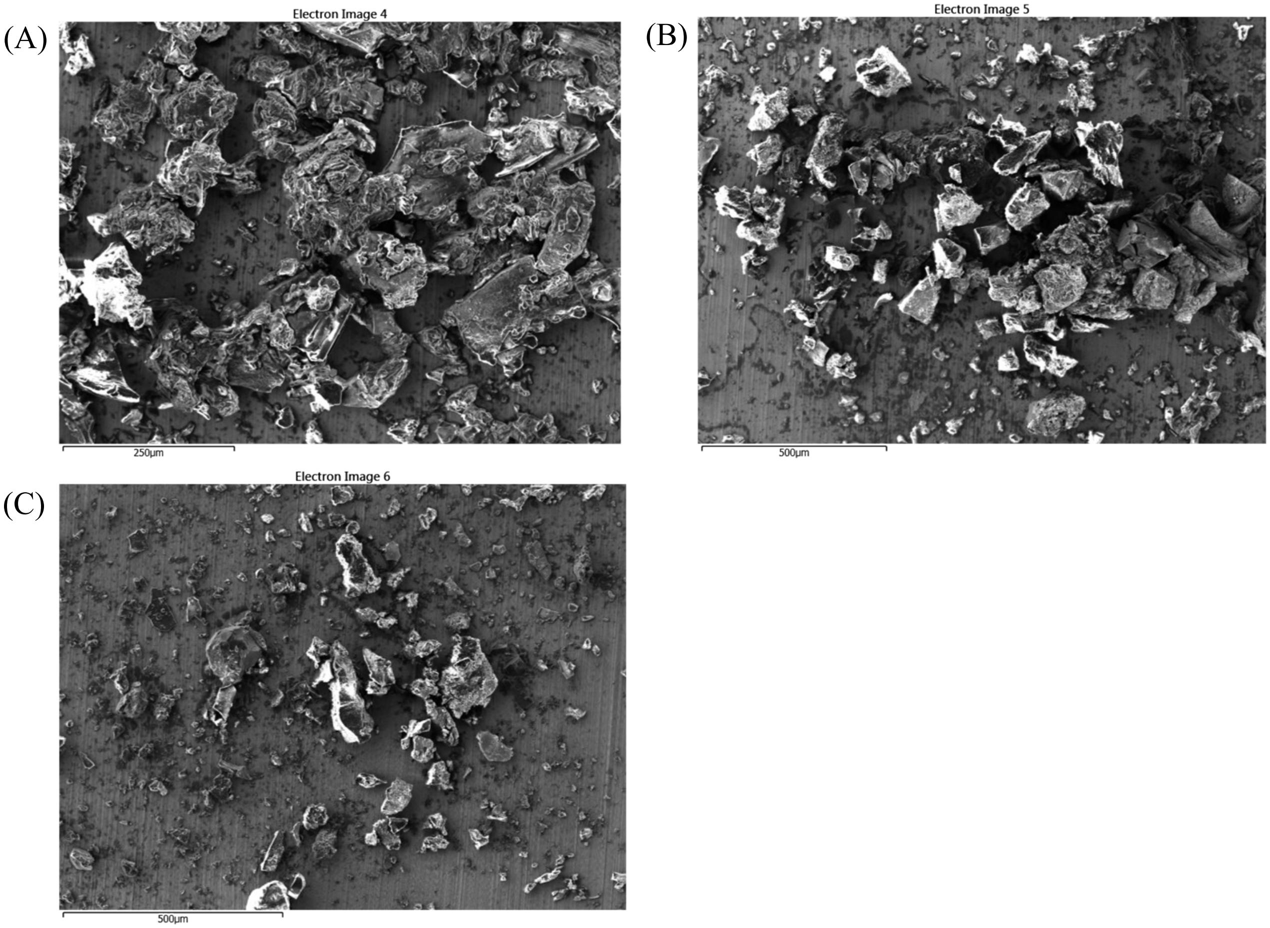

Table S2.

Element	Site 1 (% ± σ)	Site 2 (% ± σ)	Site 3 (% ± σ)	Average (± STD)
C	45.7 ± 0.2%	44.0 ± 0.2%	41.5 ± 0.2%	43.7 ± 0.3%
N	23.6 ± 0.3%	21.8 ± 0.3%	24.2 ± 0.3%	23.2 ± 0.5%
O	30.1 ± 0.2%	34.0 ± 0.2%	33.2 ± 0.2%	32.4 ± 0.3%
Ca	0.2 ± 0.1%	---------------	0.5 ± 0.1%	0.4 ± 0.1%
Si	0.2 ± 0.0%	---------------	0.2 ± 0.0%	0.2 ± 0.0%
Cl	0.2 ± 0.0%	0.3 ± 0.1%	0.3 ± 0.1%	0.3 ± 0.1%

100sec MW CDs EDS data. Calcium, silicon, and chlorine are considered as contaminants and not additional dopants since they were not present in the starting materials.

Section D – ATR-FTIR spectra

Figure S18. Oven CDs ATR-FTIR spectrum.

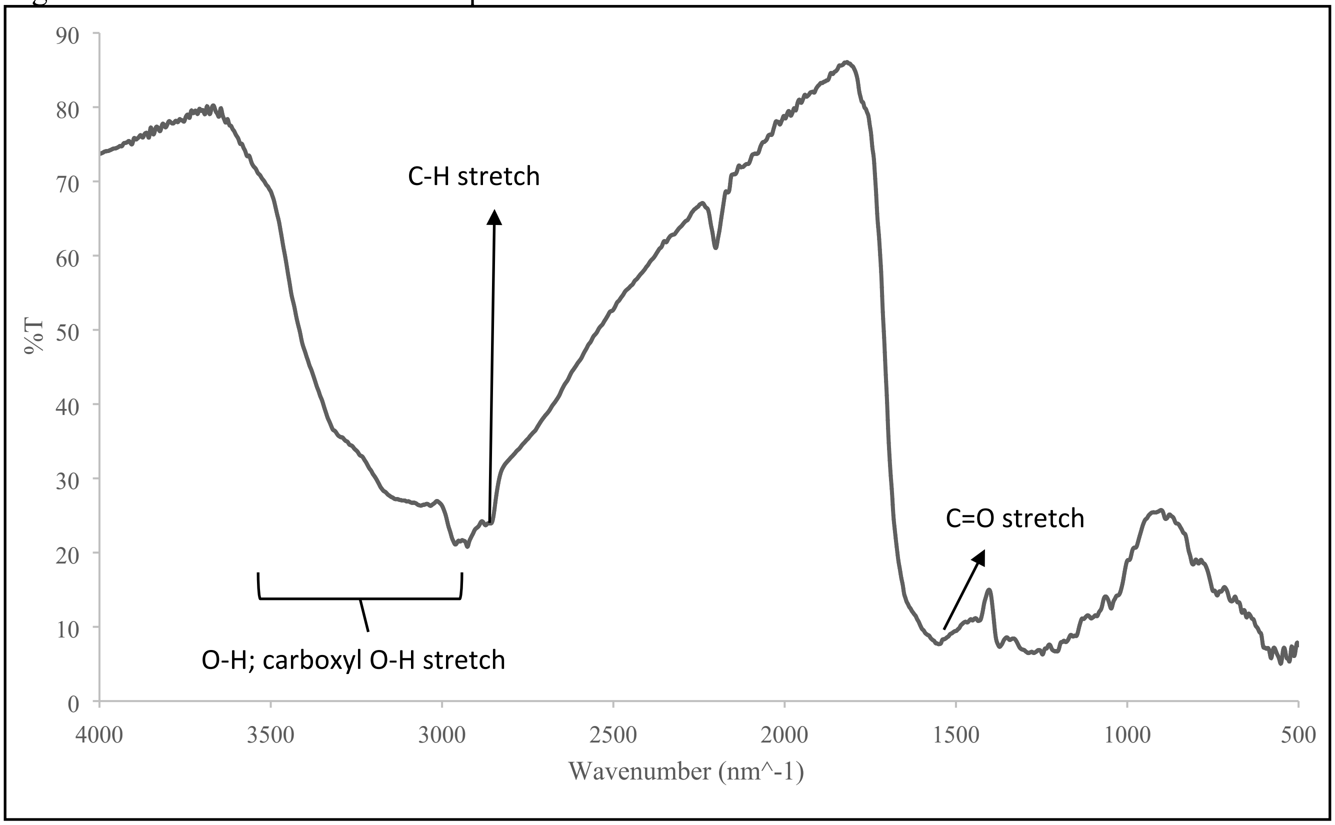

Figure S19. 100sec MW CDs ATR-FTIR spectrum.

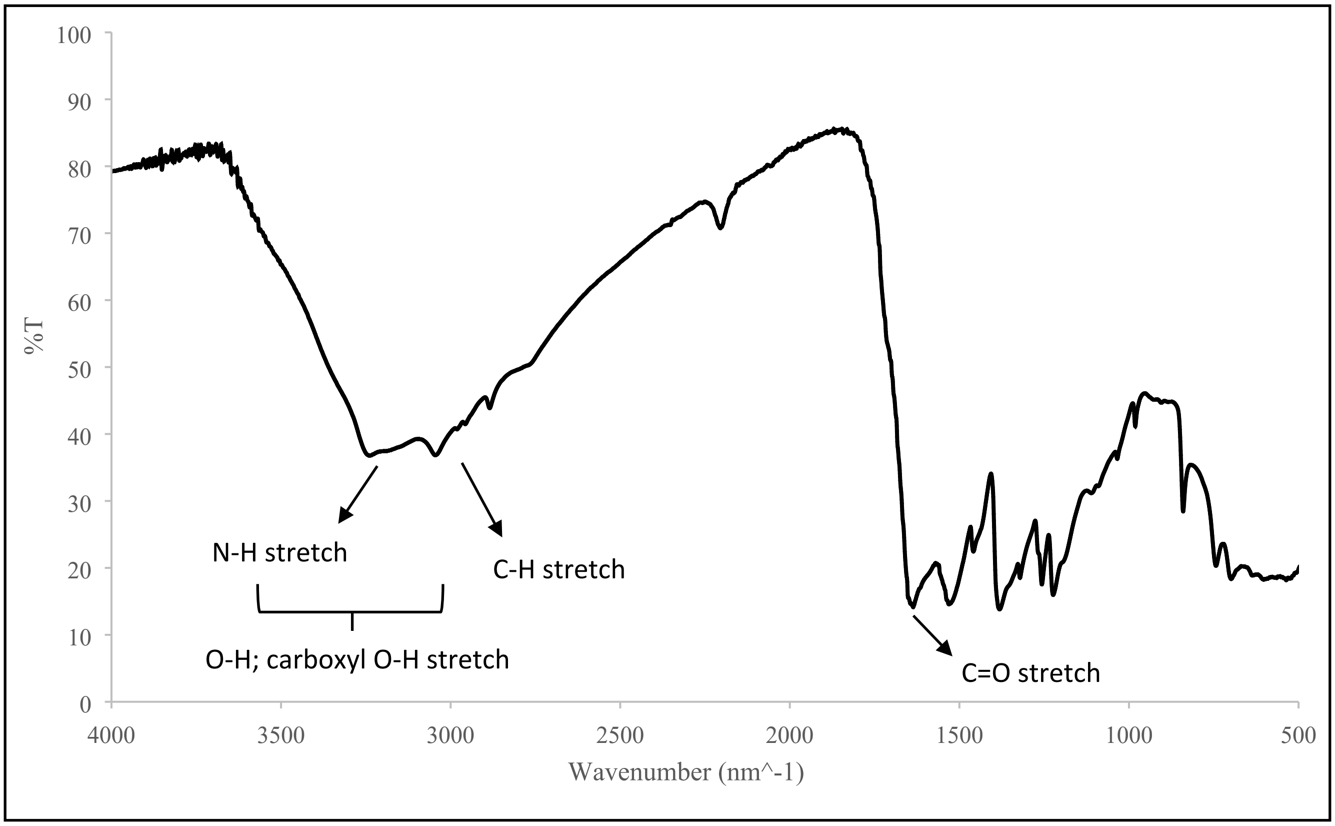

Section E – Peak Fluorescence Measurements Specifications

Table S3.

CDs Solution Type	Solution Concentration (% CDs solution in solvent)	Fixed Excitation Wavelength for Emission Scan (nm)	Fixed Emission Wavelength for Excitation Scan (nm)
Oven in Formamide	5%	410	475
MW 100sec in Formamide	17.5%	418	475
MW 80sec in Formamide	50%	420	484
MW 60sec in Formamide	100%	419	495
MW 40sec in Formamide	100%	420	470
Oven in DI Water	10%	402	477
MW 100sec in DI Water	75%	375	459
MW 80sec in DI Water	100%	376	455
MW 60sec in DI Water	100%	376	458
MW 40sec in DI Water	100%	375	458
Oven in PBS	10%	405	470
MW 100sec in PBS	50%	405	470
MW 80sec in PBS	50%	384	463
MW 60sec in PBS	100%	384	462
MW 40sec in PBS	100%	370	455

Section F – Lifetimes Data

Each type of CDs solution in PBS was analyzed by determining fluorescence lifetime decay measurements through a single photon counting controller and spectrometer. From the raw data, τ (lifetime decay) was ultimately calculated using Equation 1.

$$\tau = \frac{1}{\lambda} \quad \text{[Equation 1]}$$

Figure S20. Oven CDs in PBS (10% concentration) emitted photon counts (intensity) versus number of channels. Emission set as 470 nm to match peak emission recorded previously.

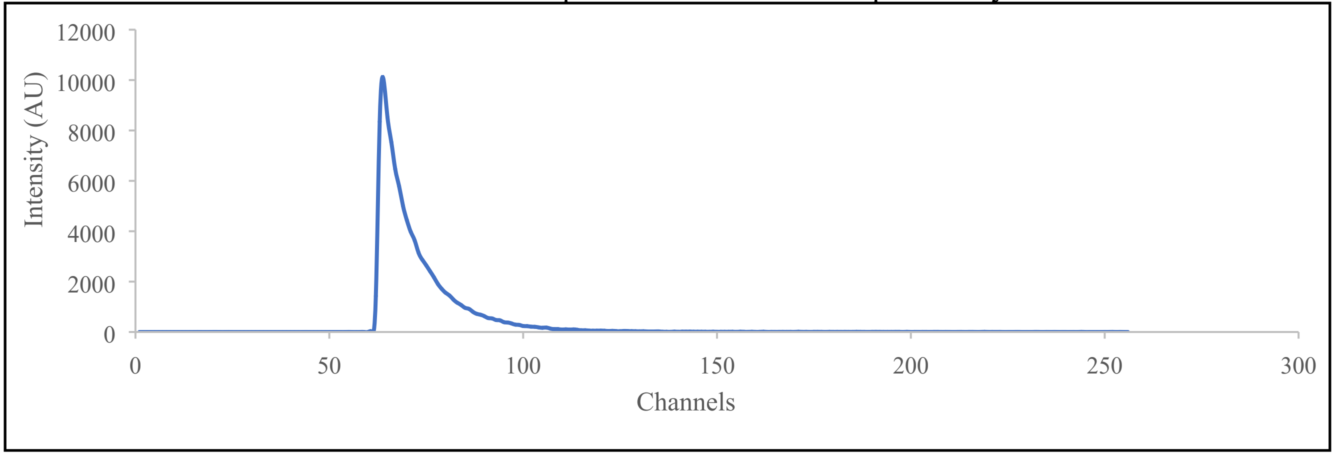

$$\frac{[y - axis\ peak\ value]}{\frac{1}{e}} = \frac{10004}{2.72} = 3677.9$$

Find data point whose x-value approximates 3677.9 → (72, 3642)

$[x - value\ at\ y = 3642] - [x - value\ at\ peak\ y - value] = 72 - 64 = 8$ channels

Instrument time calibration = $0.8779 \frac{ns}{channel}$

$8\ channels * 0.8779 \frac{ns}{channel} = 7.023$ **ns lifetime decay**

Figure S21. 60sec MW CDs in PBS (100% concentration) emitted photon counts (intensity) versus number of channels. Emission set as 462nm to match peak emission recorded previously.

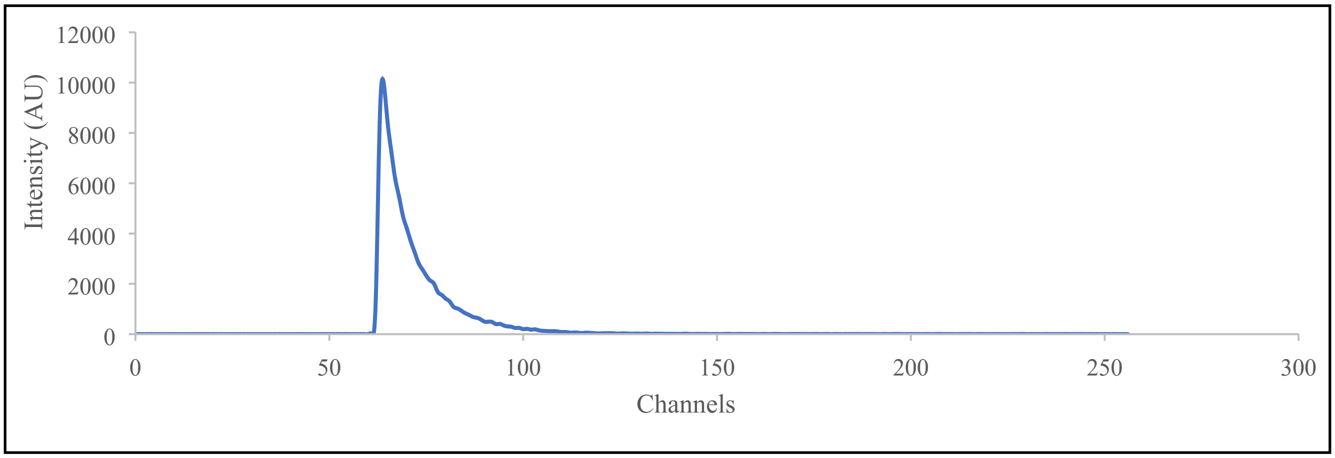

$$\frac{[y - axis\ peak\ value]}{\frac{1}{e}} = \frac{10005}{2.72} = 3678.3$$

Find data point whose x-value approximates 3678.3 → (71, 3693)

$$[x - value\ at\ y = 3693] - [x - value\ at\ peak\ y - value] = 71 - 64 = 7$$ channels

Instrument time calibration = $0.8779\frac{ns}{channel}$

$$7\ channels * 0.8779\frac{ns}{channel} = 6.145$$ **ns lifetime decay**

<u>Section G – Cell Sample Preparation Methodology for CDs Toxicity Experimentation</u>

The following diagram portrays the steps involved for sample preparation for cell toxicity experiments conducted with HL60 cells. Control and test samples were processed in the same manner, only differing in the composition of their staining solution, so that they would experience the same amount of stress. Unlike the test sample, the cells in the control sample would not appear black as they do in the fourth step of this process.

Figure S22.

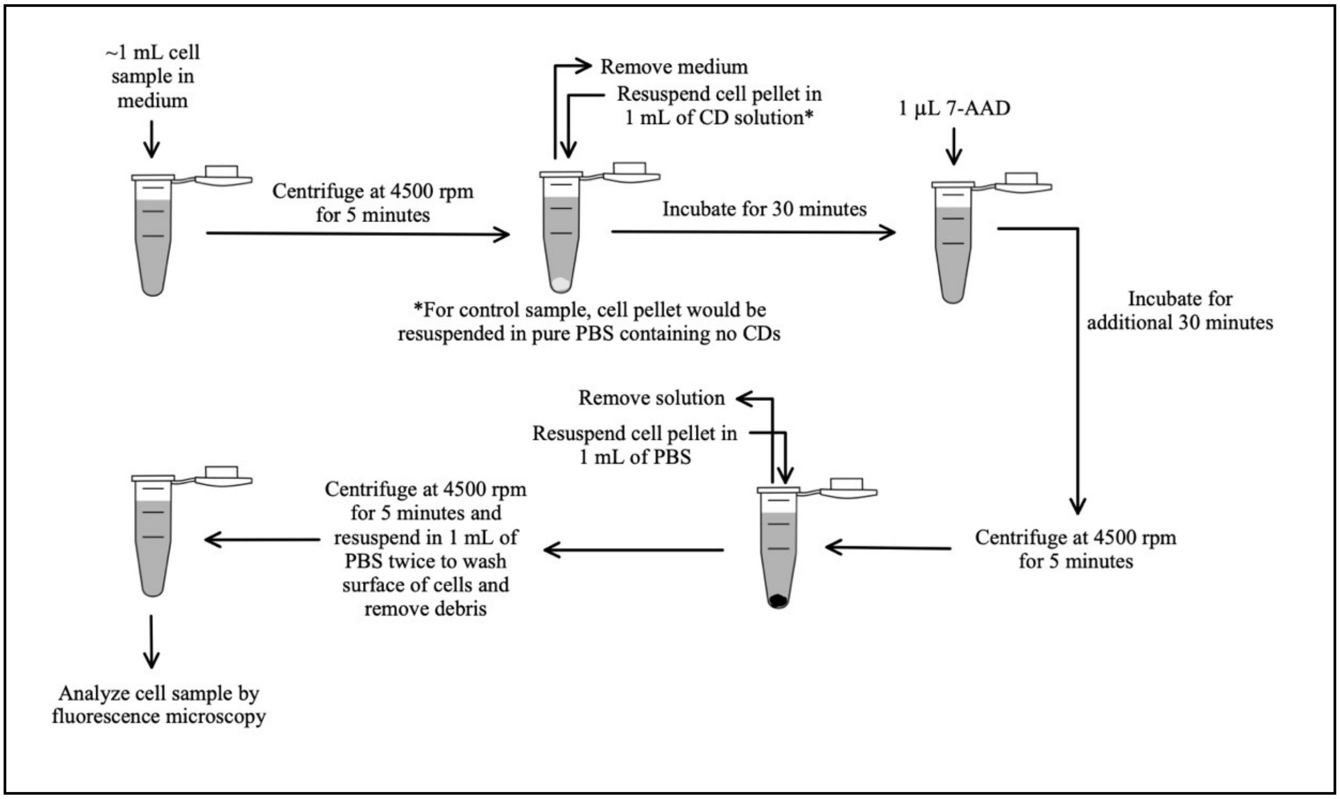

Section H – Biocompatibility Experimental Microscopic Images (Representative)

Microscopic images were captured with 20X objective under white light, blue-light bandpass filter, green-light bandpass filter, and red-light bandpass filter to visualize the condition of cells utilized for biocompatibility experiments.

Figure S23. Representative control sample images consisting of HL60 cells in PBS stained with 7-AAD dye. (A) White light image, (B) blue-light bandpass filtered image, (C) green-light bandpass filtered image, and (D) red-light bandpass filtered image.

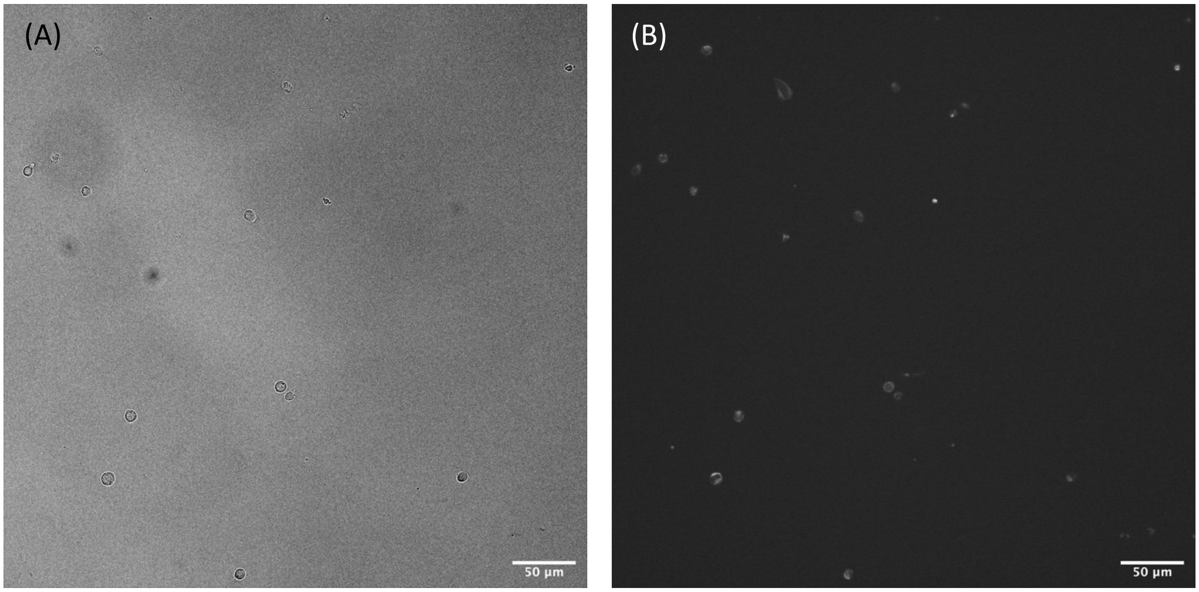

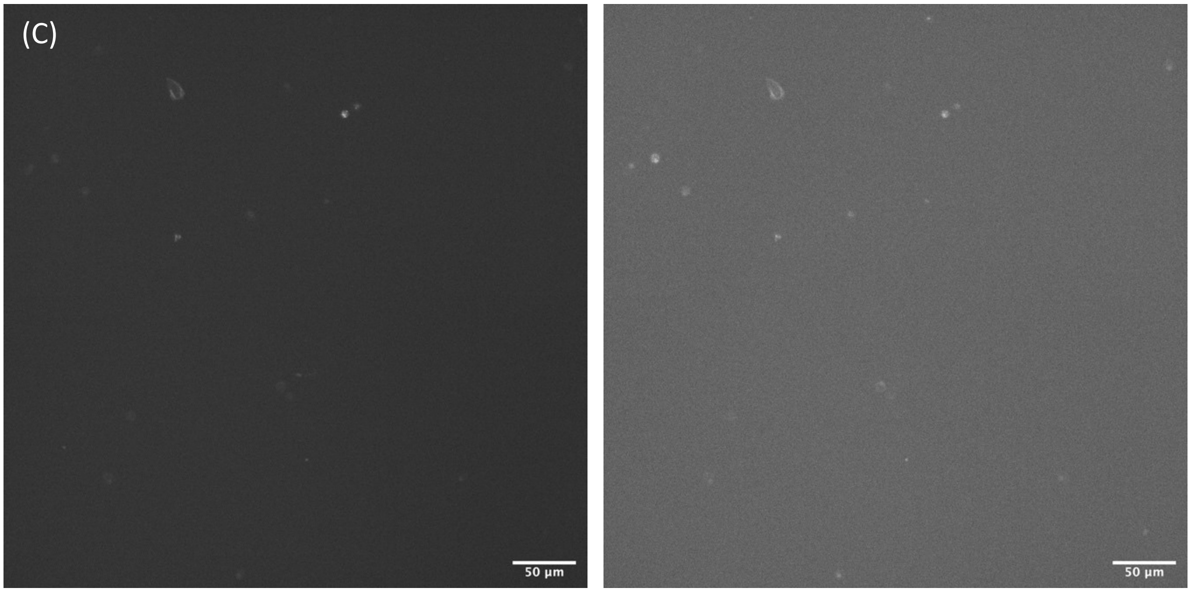

Figure S24. Representative cell sample images consisting of HL60 cells stained with a 10% oven CDs solution in PBS and co-stained with 7-AAD dye. (A) White light image, (B) blue-light bandpass filtered image, (C) green-light bandpass filtered image, and (D) red-light bandpass filtered image.

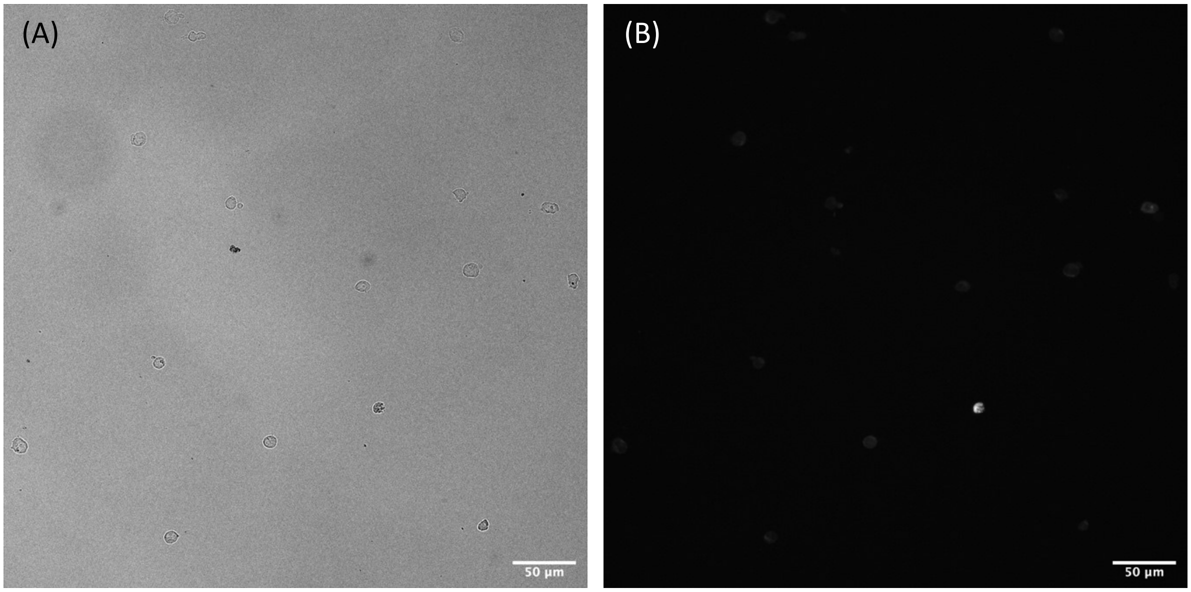

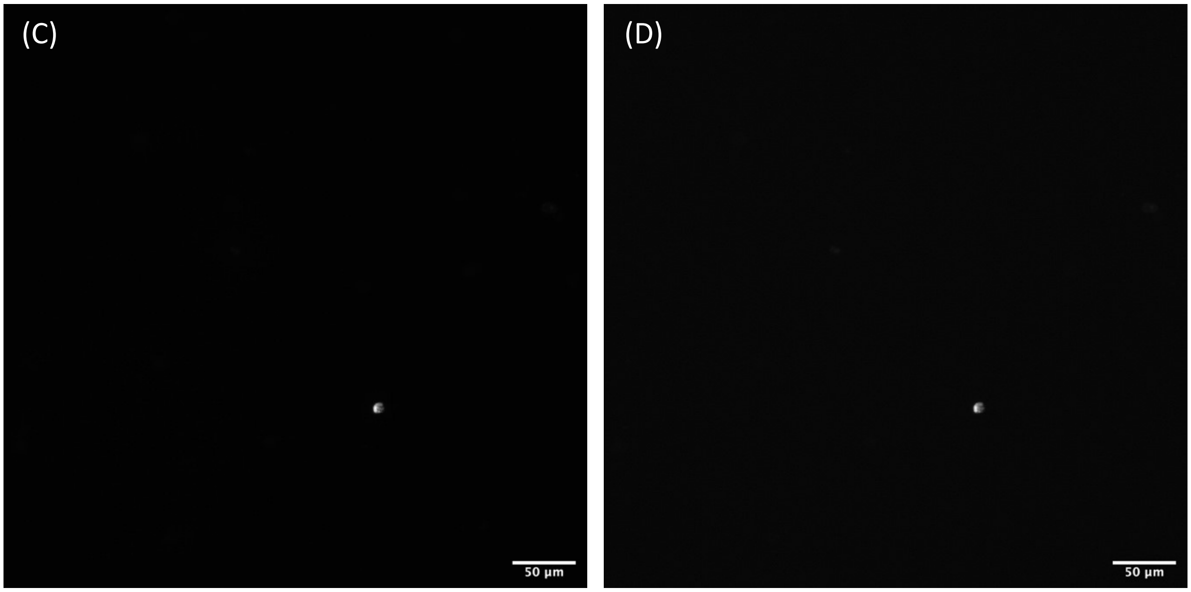

Figure S25. Representative cell sample images consisting of HL60 cells stained with a 50% 60sec MW CDs solution in PBS and co-stained with 7-AAD dye. (A) White light image, (B) blue-light bandpass filtered image, (C) green-light bandpass filtered image, and (D) red-light bandpass filtered image.

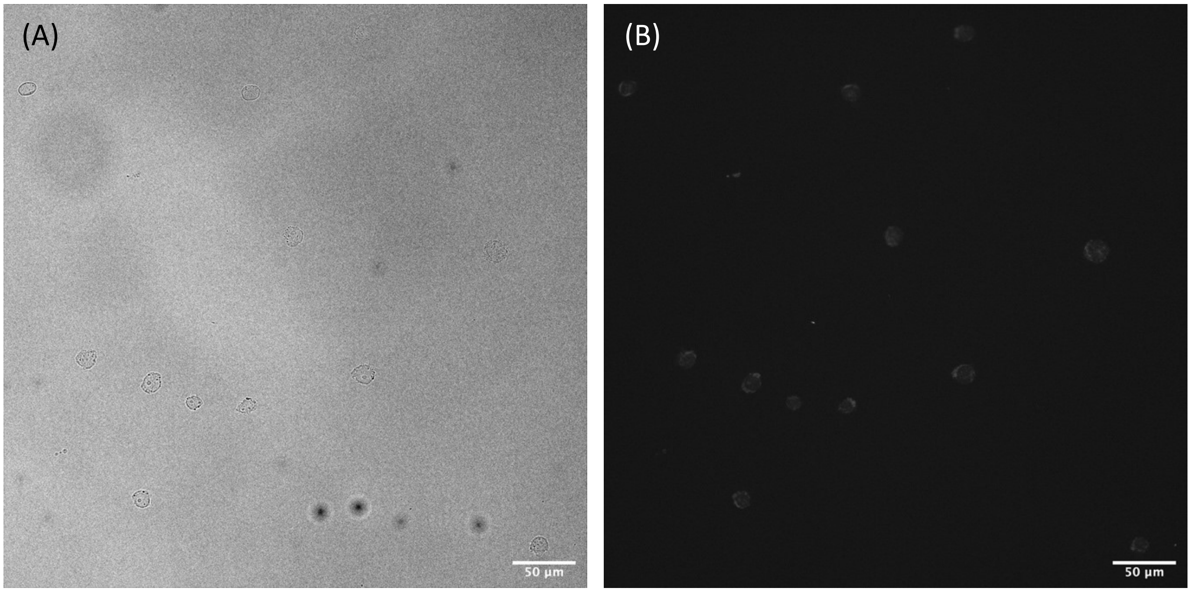

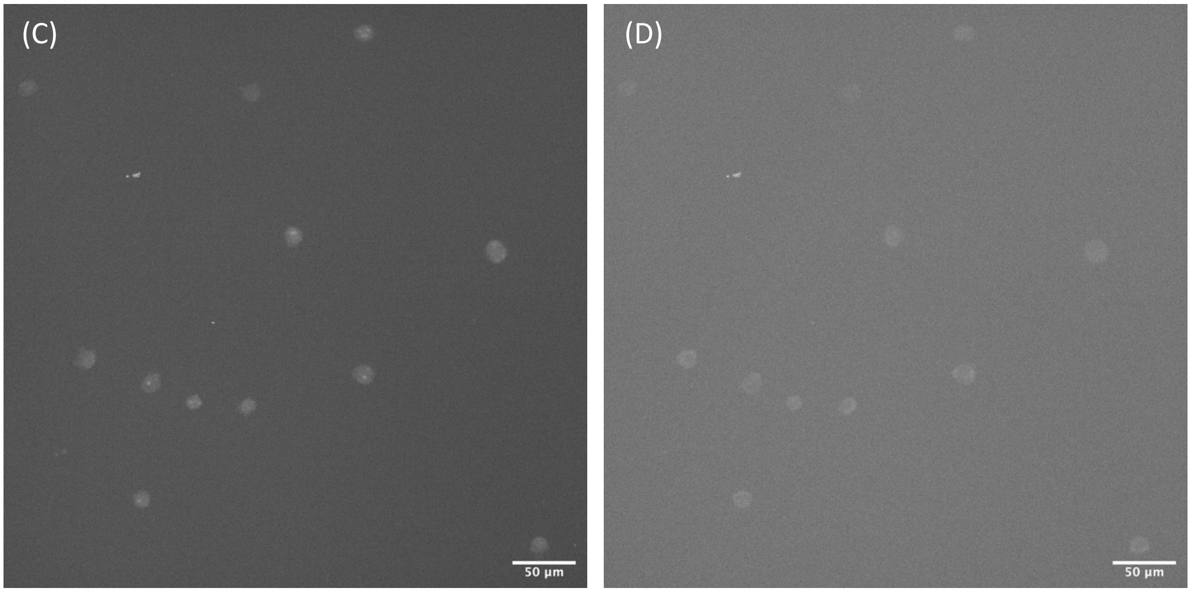

Section I – Biocompatibility Experimental Flow Cytometry Histograms

Figure S26. Control HL60 cells (no CDs, PBS and 7-AAD dye only) fluorescence intensity distribution. Gated region "M1" represents the subpopulation of dead cells while "M2" represents the subpopulation of live cells (the value with which cell samples were compared by t-test). The dead cells have high fluorescence intensity due to staining by 7-AAD (gates previously established through cell sample treated with ethanol) while the live cells have a comparatively lower intensity due to autofluorescence. Measurements were obtained in triplicate: (A) Trial 1, (B) Trial 2, and (C) Trial 3.

(A)

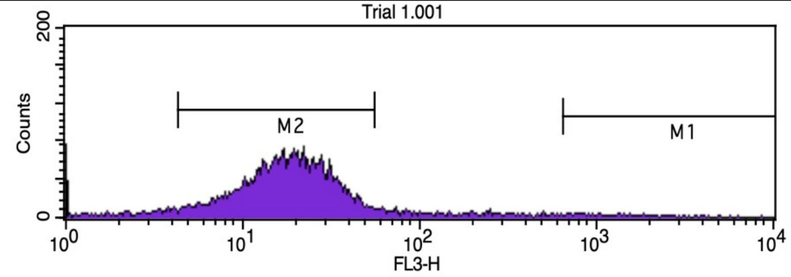

Histogram Statistics

File: Trial 1.001
Sample ID:
Tube: Untitled
Acquisition Date: 29-Jan-71
Gated Events: 10000
X Parameter: FL3-H (Log)

Log Data Units: Linear Values
Patient ID:
Panel: Untitled Acquisition Tube List
Gate: No Gate
Total Events: 10000

Marker	Left, Right	Events	% Gated	% Total	Mean	Geo Mean	CV	Median	Peak Ch
All	1, 9910	10000	100.00	100.00	62.86	20.04	424.21	18.77	1
M1	649, 9910	224	2.24	2.24	1490.80	1292.45	65.57	1229.83	697
M2	4, 55	8657	86.57	86.57	20.00	17.50	50.69	18.11	21

(B)

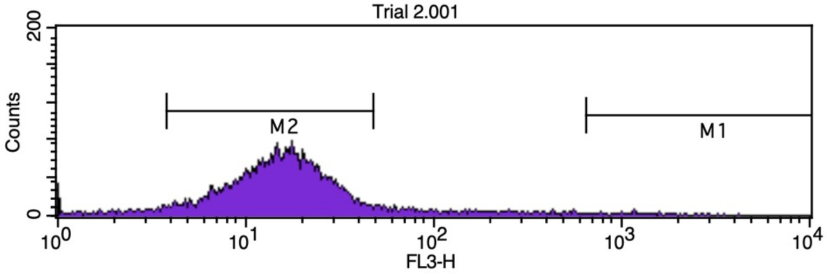

Histogram Statistics

File: Trial 2.001
Sample ID:
Tube: Untitled
Acquisition Date: 29-Jan-71
Gated Events: 10000
X Parameter: FL3-H (Log)

Log Data Units: Linear Values
Patient ID:
Panel: Untitled Acquisition Tube List
Gate: No Gate
Total Events: 10000

Marker	Left, Right	Events	% Gated	% Total	Mean	Geo Mean	CV	Median	Peak Ch
All	1, 9910	10000	100.00	100.00	42.39	17.02	374.54	15.96	17
M1	649, 9910	121	1.21	1.21	1239.10	1129.73	49.97	1046.00	1124
M2	4, 47	8739	87.39	87.39	16.92	14.84	50.73	15.40	17

(C)

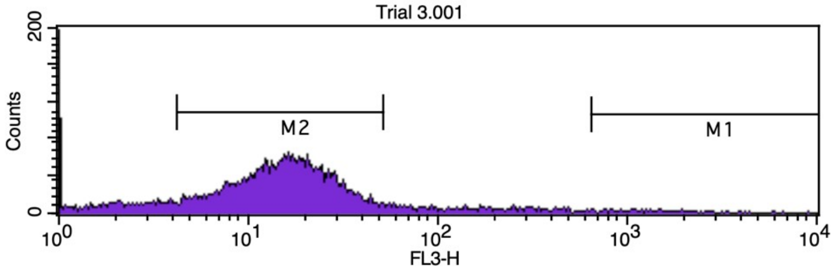

Histogram Statistics

File: Trial 3.001
Sample ID:
Tube: Untitled
Acquisition Date: 29-Jan-71
Gated Events: 10000
X Parameter: FL3-H (Log)

Log Data Units: Linear Values
Patient ID:
Panel: Untitled Acquisition Tube List
Gate: No Gate
Total Events: 10000

Marker	Left, Right	Events	% Gated	% Total	Mean	Geo Mean	CV	Median	Peak Ch
All	1, 9910	10000	100.00	100.00	55.48	15.13	455.03	15.12	1
M1	649, 9910	183	1.83	1.83	1520.37	1315.88	69.08	1165.22	791
M2	4, 51	7903	79.03	79.03	17.34	15.03	53.46	15.54	15

Figure S27. Oven CDs-stained HL60 cells (10% concentration of CDs in PBS and co-stained with 7-AAD dye) fluorescence intensity distribution. Gated region "M1" represents the subpopulation of dead cells while "M2" represents the subpopulation of live cells (the value with which cell samples were compared by t-test). The dead cells have high fluorescence intensity due to staining by 7-AAD (gates previously established through cell sample treated with ethanol) while the live cells have a comparatively lower intensity due to CDs fluorescence. Measurements were obtained in triplicate: (A) Trial 1, (B) Trial 2, and (C) Trial 3.

(A)

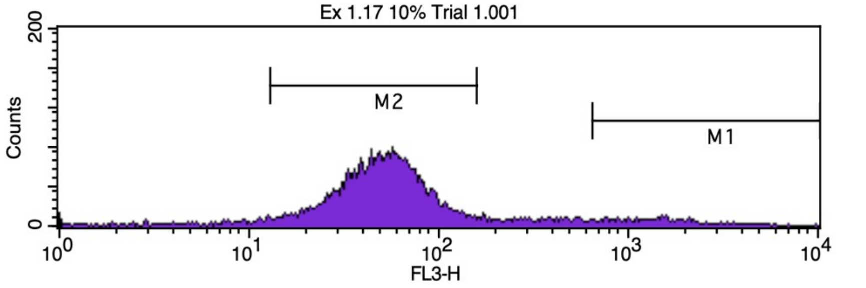

Histogram Statistics

File: Ex 1.17 10% Trial 1.001
Sample ID:
Tube: Untitled
Acquisition Date: 29-Jan-71
Gated Events: 10000
X Parameter: FL3-H (Log)

Log Data Units: Linear Values
Patient ID:
Panel: Untitled Acquisition Tube List
Gate: No Gate
Total Events: 10000

Marker	Left, Right	Events	% Gated	% Total	Mean	Geo Mean	CV	Median	Peak Ch
All	1, 9910	10000	100.00	100.00	167.54	62.84	277.24	53.28	55
M1	649, 9910	616	6.16	6.16	1623.74	1410.18	65.59	1345.57	1539
M2	13, 158	8442	84.42	84.42	54.87	48.97	48.24	50.03	55

(B)

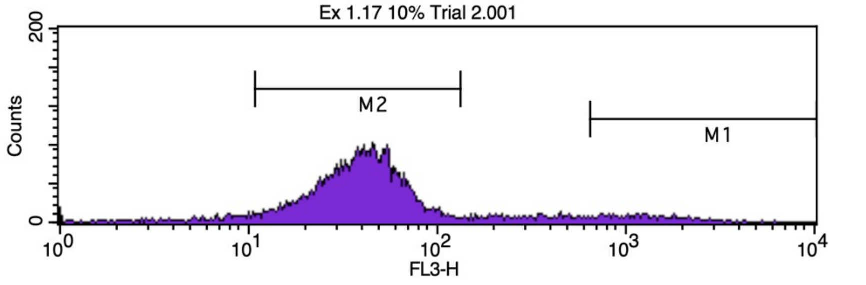

Histogram Statistics

File: Ex 1.17 10% Trial 2.001
Sample ID:
Tube: Untitled
Acquisition Date: 29-Jan-71
Gated Events: 10000
X Parameter: FL3-H (Log)

Log Data Units: Linear Values
Patient ID:
Panel: Untitled Acquisition Tube List
Gate: No Gate
Total Events: 10000

Marker	Left, Right	Events	% Gated	% Total	Mean	Geo Mean	CV	Median	Peak Ch
All	1, 9910	10000	100.00	100.00	132.99	48.58	276.79	41.79	44
M1	649, 9910	537	5.37	5.37	1423.00	1275.31	56.43	1207.90	827
M2	11, 132	8469	84.69	84.69	42.58	38.24	46.75	39.60	44

(C)

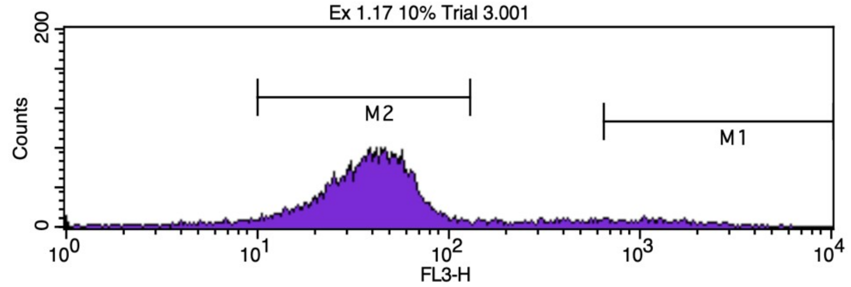

Histogram Statistics

File: Ex 1.17 10% Trial 3.001
Sample ID:
Tube: Untitled
Acquisition Date: 29-Jan-71
Gated Events: 10000
X Parameter: FL3-H (Log)

Log Data Units: Linear Values
Patient ID:
Panel: Untitled Acquisition Tube List
Gate: No Gate
Total Events: 10000

Marker	Left, Right	Events	% Gated	% Total	Mean	Geo Mean	CV	Median	Peak Ch
All	1, 9910	10000	100.00	100.00	126.37	46.49	281.32	40.32	37
M1	649, 9910	505	5.05	5.05	1420.83	1264.70	55.13	1154.78	1027
M2	10, 129	8581	85.81	85.81	41.30	37.04	46.65	38.54	37

Figure S28. 60sec MW CDs-stained HL60 cells (50% concentration of CDs in PBS and co-stained with 7-AAD dye) fluorescence intensity distribution. Gated region "M1" represents the subpopulation of dead cells while "M2" represents the subpopulation of live cells (the value with which cell samples were compared by t-test). The dead cells have high fluorescence intensity due to staining by 7-AAD (gates previously established through cell sample treated with ethanol) while the live cells have a comparatively lower intensity due to CDs fluorescence. Measurements were obtained in triplicate: (A) Trial 1, (B) Trial 2, and (C) Trial 3.

(A)

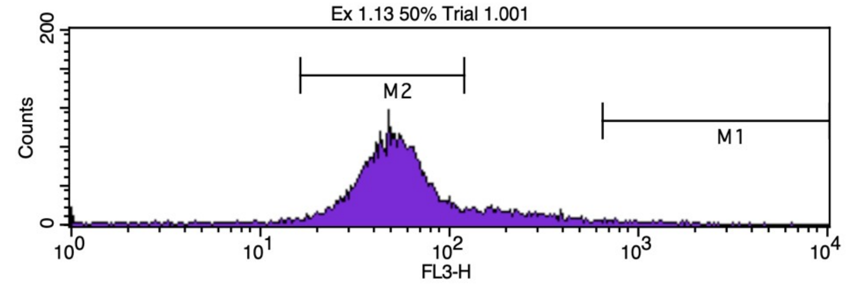

Histogram Statistics

File: Ex 1.13 50% Trial 1.001
Sample ID:
Tube: Untitled
Acquisition Date: 29-Jan-71
Gated Events: 10000
X Parameter: FL3-H (Log)

Log Data Units: Linear Values
Patient ID:
Panel: Untitled Acquisition Tube List
Gate: No Gate
Total Events: 10000

Marker	Left, Right	Events	% Gated	% Total	Mean	Geo Mean	CV	Median	Peak Ch
All	1, 9910	10000	100.00	100.00	93.83	58.85	197.30	52.80	47
M1	649, 9910	150	1.50	1.50	1245.41	1121.48	58.24	1022.74	736
M2	16, 119	8302	83.02	83.02	52.27	48.71	37.89	49.58	47

(B)

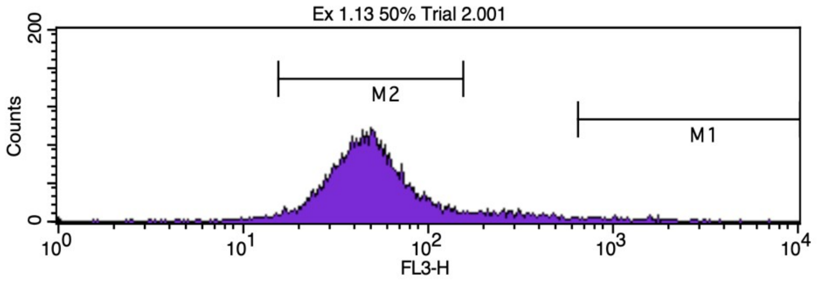

Histogram Statistics

File: Ex 1.13 50% Trial 2.001
Sample ID:
Tube: Untitled
Acquisition Date: 29-Jan-71
Gated Events: 10000
X Parameter: FL3-H (Log)

Log Data Units: Linear Values
Patient ID:
Panel: Untitled Acquisition Tube List
Gate: No Gate
Total Events: 10000

Marker	Left, Right	Events	% Gated	% Total	Mean	Geo Mean	CV	Median	Peak Ch
All	1, 9910	10000	100.00	100.00	90.09	54.43	225.65	47.83	48
M1	649, 9910	178	1.78	1.78	1281.69	1148.03	58.86	1031.98	982
M2	15, 154	8917	89.17	89.17	51.11	46.33	47.31	45.73	48

(C)

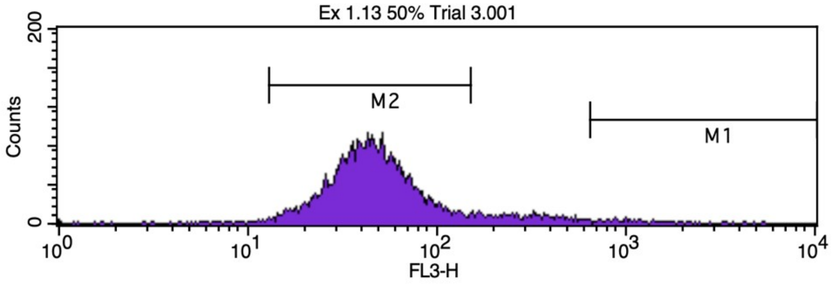

Histogram Statistics

File: Ex 1.13 50% Trial 3.001
Sample ID:
Tube: Untitled
Acquisition Date: 29-Jan-71
Gated Events: 10000
X Parameter: FL3-H (Log)

Log Data Units: Linear Values
Patient ID:
Panel: Untitled Acquisition Tube List
Gate: No Gate
Total Events: 10000

Marker	Left, Right	Events	% Gated	% Total	Mean	Geo Mean	CV	Median	Peak Ch
All	1, 9910	10000	100.00	100.00	88.00	52.09	254.43	45.73	42
M1	649, 9910	166	1.66	1.66	1322.23	1133.15	80.13	991.05	842
M2	13, 150	8990	89.90	89.90	48.76	43.88	48.50	43.71	42
